# Enhanced Effects of Complex Tea Extract and the Postbiotic BPL1^®^ HT on Ameliorating the Cardiometabolic Alterations Associated with Metabolic Syndrome in Mice

**DOI:** 10.3390/ijms27020680

**Published:** 2026-01-09

**Authors:** Mario de la Fuente-Muñoz, Marta Román-Carmena, Sara Amor, Daniel González-Hedström, Verónica Martinez-Rios, Sonia Guilera-Bermell, Francisco Canet, Araceli Lamelas, Ángel Luis García-Villalón, Patricia Martorell, Antonio M. Inarejos-García, Miriam Granado

**Affiliations:** 1 Physiology Department, School of Medicine, Universidad Autonoma de Madrid, 28029 Madrid, Spainmarta.romanc@uam.es (M.R.-C.); sara.amor@uam.es (S.A.); angeluis.villalon@uam.es (Á.L.G.-V.); 2 ADM Research Center-Valencia, Universitat de València, 46980 Paterna, Spain; daniel.gonzalezhedstrom@adm.com (D.G.-H.); veronica.martinez@adm.com (V.M.-R.); sonia.guilera@adm.com (S.G.-B.); patricia.martorell@adm.com (P.M.); antonio.inarejos@adm.com (A.M.I.-G.); 3 CIBER Fisiopatología de la Obesidad y Nutrición, Instituto de Salud Carlos III, 28029 Madrid, Spain

**Keywords:** metabolic syndrome, insulin resistance, hypertension, nutritional supplements, tea extract, microbiota, postbiotic

## Abstract

Metabolic syndrome (MetS) is a multifactorial disorder characterized by central obesity, insulin resistance, dyslipidemia, and hypertension, all of which increase the risk of type 2 diabetes and cardiovascular diseases. This study investigates the potential complementary effects of the standardized green and black ADM ComplexTea Extract (CTE) and the heat-treated postbiotic (BPL1^®^ HT) on the cardiometabolic alterations associated with MetS in a murine model. C57BL/6J mice were fed a high-fat/high-sucrose (HFHS) diet and treated with CTE, BPL1^®^ HT, or their combination for 20 weeks. Metabolic, inflammatory, oxidative, vascular parameters, and fecal microbiota composition were assessed. Both CTE and BPL1^®^ HT individually attenuated weight gain, organ hypertrophy, insulin resistance, and inflammation. However, their combined administration exerted synergistic effects, fully normalizing body weight, adipocyte size, lipid profiles, HOMA-IR index, and insulin sensitivity to levels comparable to lean controls. Co-treatment also restored PI3K/Akt signaling in liver and muscle, reduced hepatic steatosis, and normalized the expression of inflammatory and oxidative stress markers across multiple tissues. Furthermore, vascular function was significantly improved, with enhanced endothelium-dependent relaxation and reduced vasoconstrictor responses, particularly to angiotensin II. CTE, BPL1^®^HT, and the blend prevented bacterial richness reduction caused by HFHS; the blend achieved higher bacterial richness than mice in Chow diet. Additionally, the blend prevented the increase in *Flintibacter butyricus*, which is associated with MetS clinical parameters, and showed a tendency to increase the abundance of *Bifidobacterium*. These findings suggest that the combination of CTE and BPL1^®^ HT offers a potential nutritional strategy to counteract the metabolic and cardiovascular complications of MetS through complementary mechanisms involving improved insulin signaling, reduced inflammation and oxidative stress, enhanced vascular function, and modulation of gut microbiota.

## 1. Introduction

Metabolic syndrome (MetS) is a multifactorial disorder characterized by a cluster of metabolic and cardiovascular abnormalities, including central obesity, dyslipidemia, insulin resistance, and hypertension, which collectively increase the risk of suffering from metabolic diseases such as type 2 diabetes and cardiovascular diseases in the long term [1].

Central to the pathogenesis of MetS is insulin resistance, a condition characterized by the disruption of the phosphatidylinositol 3-kinase/protein kinase B (PI3K/Akt) signaling pathway, a critical mediator of insulin’s metabolic actions in key metabolic tissues such as liver, muscle, and adipose tissue [2,3]. Under physiological conditions, insulin binding to its receptor initiates a cascade involving insulin receptor substrate (IRS) proteins, PI3K activation, and subsequent Akt phosphorylation, ultimately promoting metabolic effects such as glucose uptake, glycogen synthesis, lipogenesis, and inhibition of lipolysis [4]. Impairment at any step, such as decreased IRS phosphorylation or PI3K/Akt activity, leads to defective glucose transport, reduced glycogen synthesis, increased lipolysis, and the development of insulin resistance [5].

One of the most important factors contributing to the development of insulin resistance is increased adiposity, and particularly visceral adiposity [6], a condition that is present in most patients with MetS [7]. Visceral adiposity is strongly correlated with elevated HOMA-IR scores, indicating increased insulin resistance and a greater risk for metabolic syndrome, even independently of overall obesity [8]. The excess release of free fatty acids and lipid metabolites by adipose tissue, such as diacylglycerols, are reported to further disrupt glucose metabolism by interfering with IRS and PI3K signaling, contributing to glucose intolerance and hyperinsulinemia and perpetuating a cycle of metabolic disruption [9,10,11]. For this reason, a great effort is being made to search for new strategies that help reduce visceral adiposity with the aim of reducing the impact of cardiometabolic alterations associated with MetS.

In addition to insulin resistance, MetS is also associated with multiple cardiovascular alterations. The cardiovascular component of MetS, particularly hypertension, is driven by a combination of endothelial dysfunction, hyperactivation of the sympathetic nervous system, increased expression of vasoconstrictors like endothelin-1, and overstimulation of the renin–angiotensin–aldosterone system (RAAS) [12]. Endothelial dysfunction, often a consequence of impaired PI3K/Akt signaling at the vascular level [13], leads to reduced nitric oxide bioavailability and increased oxidative stress, further promoting vascular stiffness and hypertension [14]. Simultaneously, increased sympathetic tone and upregulation of vasoconstrictors contribute to increased vascular resistance, while RAAS hyperactivation exacerbates sodium retention and vascular remodeling, collectively elevating blood pressure [12].

Inflammation and oxidative stress play critical roles in the development of both insulin resistance and hypertension within the context of MetS. In this regard, oxidative stress and inflammation have been shown to precede and predict insulin resistance and vascular abnormalities in experimental models of MetS, suggesting that they are primary drivers rather than consequences of cardiometabolic dysfunction [15]. Indeed, the synergistic interaction between these two processes is now considered a central mechanism in the pathophysiology of metabolic syndrome [16]. Chronic low-grade inflammation, often originating from adipose tissue, leads to increased production of cytokines like TNF-α and IL-6, which impair insulin receptor signaling and contribute to insulin resistance [6]. Simultaneously, oxidative stress from reactive oxygen species (ROS) damages cellular structures and signaling pathways essential for insulin sensitivity [17]. Particularly, excessive production of ROS disrupts insulin signaling by activating stress-sensitive kinases, such as IKK-β, which phosphorylate key proteins in the insulin signaling pathway (e.g., insulin receptor substrate proteins), ultimately impairing glucose uptake in peripheral tissues [18]. In parallel, oxidative stress activates inflammatory pathways, leading to the release of proinflammatory cytokines and adhesion molecules, which further exacerbate insulin resistance and create a chronic low-grade inflammatory state [19]. In the vascular endothelium, ROS activates the renin–angiotensin system [20] and reduces nitric oxide (NO) bioavailability by promoting NO inactivation and uncoupling of endothelial nitric oxide synthase (eNOS), resulting in impaired vasodilation and endothelial dysfunction [21]. This dysfunction not only contributes to the progression of hypertension but also perpetuates a cycle of oxidative stress and inflammation, further damaging vascular and metabolic homeostasis [22]. Collectively, the interplay between oxidative stress and inflammation forms a pathogenic nexus that underlies the onset and progression of insulin resistance, endothelial dysfunction, and hypertension in the context of obesity and MetS [23].

On the other hand, the gut microbiota plays a crucial role in the development and progression of MetS by influencing host metabolism, immune responses, and energy balance [24,25]. Dysbiosis, an imbalance in the gut microbial community, has been linked to increased intestinal permeability, metabolic endotoxemia, chronic low-grade inflammation, and insulin resistance, all of which contribute to MetS [24]. In this regard, products that beneficially impact the gut microbiota, such as probiotics and prebiotics, have shown promise in restoring microbial diversity, enhancing the production of beneficial metabolites like short-chain fatty acids, and reducing inflammation, thereby ameliorating metabolic disturbances associated with MetS [25]. Another type of product that impacts microbiota and is less well-known than the previous ones are postbiotics. Postbiotics are defined by the International Scientific Association of Probiotics and Prebiotics (ISAPP) as preparations of inanimate microorganisms and/or their components that confer a health benefit on the host [26] and have arose as promising candidates to manage the co-morbidities associated with MetS including obesity, insulin resistance/type 2 diabetes, and non-alcoholic fatty liver disease [27]. Indeed, in some cases, these beneficial effects of inanimate microorganisms seem to be more evident than those exerted by live microorganisms [28].

The growing prevalence of MetS underscores the urgent need for novel preventive and therapeutic strategies. In this regard, recent research has highlighted the potential of bioactive compounds from natural sources and microbiota-targeted interventions to ameliorate key features of this syndrome, such as metabolic and cardiovascular disturbances. Tea extracts, rich in bioactive polyphenols, have demonstrated antioxidant, anti-inflammatory, and insulin-sensitizing effects, attenuating PI3K/Akt pathway function and improving endothelial health. Particularly, our group has previously reported the beneficial cardiometabolic effects of the standardized green and black tea extract ADM Tea Complex (CTE) commercialized by the company ADM in the context of metabolic syndrome [29,30] and AngII-induced hypertension [31]. On the other hand, BPL1^®^ HT, a novel postbiotic based on heat-treated BPL1 (*Bifidobacterium animalis* sub. *lactis* CECT 8145) with insulin sensitizing, antiadipogenic [32], and antihypertensive effects [33], may beneficially modulate gut microbiota and host signaling pathways, including Akt phosphorylation, thereby ameliorating insulin resistance and vascular dysfunction. Together, the aim of this study was to analyze the possible complementary effects of ADM Tea Complex (CTE) and the postbiotic BPL1^®^ HT on the management of cardiometabolic alterations associated with MS in mice.

## 2. Results

### 2.1. Bioaccesability of Bioactive Components of Complex Tea Extract

After the digestion process, Complex Tea Extract exhibited a significant reduction in flavan-3-ols and methylxanthines concentrations throughout the entire process (salivary, gastric, and intestinal steps), as shown in Table 1. Specifically, total flavan-3-ols experienced approximately a 5-fold greater decrease after digestion compared to total xanthines (15.82-fold vs. 3.64-fold reductions, respectively). Regarding individual compounds, EGCg showed higher decrease after digestion (84.45-fold reduction). In contrast, theophylline demonstrated the smallest decrease after digestion, with only a 2.50-fold reduction.

### 2.2. Impact of CTE and BPL1^®^ HT Supplementation on Body Weight Gain and Caloric Intake

The consumption of the HFHS diet was associated with a significant increase in body weight gain (Table 2; *p* < 0.001) that was attenuated by supplementation with both CTE and BPL1^®^ HT alone (*p* < 0.01 each). Despite this reduction, body weight gain of animals from these groups was still significantly higher than body weight gain of control mice fed chow (*p* < 0.001). On the contrary, mice co-administered with both CTE and BPL1^®^ HT showed a similar body weight gain pattern to that of control animals, indicating an enhanced effect between them on body weight reduction. However, all three interventions had the same impact on caloric intake, reducing the amount of kcal ingested compared to untreated mice with MetS (*p* < 0.05 for all).

### 2.3. Impact of CTE and BPL1^®^ HT Supplementation on Body Composition

Compared to control mice fed with chow, mice with MetS showed increased weight of all the analyzed organs and tissues including the heart (*p* < 0.05), the liver (*p* < 0.05), the spleen (*p* < 0.001), the kidneys (*p* < 0.001), the adrenal glands (*p* < 0.01), the gastrocnemius muscle (*p* < 0.001), and all the adipose tissue depots: brown interscapular, brown periaortic, visceral epidydimal, visceral retroperitoneal, and inguinal subcutaneous (*p* < 0.001 for all). Some of these alterations, like the increased weights of the heart, the adrenal glands, the kidneys, or the gastrocnemius muscle, were prevented by supplementation with both CTE and BPL1^®^ HT alone (Table 2). However, the administration of each supplement separately only attenuated, but not reversed, the obesity-induced increase in the weights of the spleen and the adipose tissue depots. On the contrary, co-administration of both ingredients restored the weight of all organs, including the adipose tissue depots and the spleen weight to control levels (Table 2), suggesting their complementary effect.

### 2.4. Effect of CTE and BPL1^®^ HT Supplementation on Lipid Profile

As shown in Table 3, MetS was associated with a significant increase in the circulating levels of total cholesterol (*p* < 0.001), LDL-cholesterol (*p* < 0.05), HDL-cholesterol (*p* < 0.01), triglycerides (*p* < 0.01), and leptin (*p* < 0.001), in comparison to mice fed with chow. In mice with MetS, treatment with CTE did not have any effect on the plasma levels of total cholesterol, LDL-c, HDL-c, and triglycerides, but it significantly decreased the circulating levels of leptin (*p* < 0.05). Mice supplemented with BPL1^®^ HT alone showed similar plasma levels of LDL-c, HDL-c, and triglycerides to untreated obese mice. However, supplementation with BPL1^®^ HT significantly reduced the plasma concentrations of total cholesterol and leptin (*p* < 0.05 and *p* < 0.01, respectively) in mice fed with the HFHS diet. The combined supplementation of CTE and BPL1^®^ HT did not have any impact on the circulating levels of HDL-c. However, mice administered with the combination of CTE and BPL1^®^ HT showed reduced levels of total cholesterol and triglycerides compared to non-supplemented mice with MetS (*p* < 0.05 for both). Moreover, the circulating levels of LDL-c and leptin were reversed by the co-administration of CTE and BPL1^®^ HT (*p* < 0.001 and *p* < 0.05, respectively), with these levels showing similar values to those of lean mice. Finally, CTE supplementation attenuated the MetS-induced increase in glycerol plasma levels and the combined treatment reversed these values to control levels (*p* < 0.01).

### 2.5. Effect of CTE and BPL1^®^ HT Supplementation on the Glycemic Status

As shown in Table 3, neither CTE nor BPL1^®^ HT prevented the MetS-induced increase in glycaemia when administered separately. However, both interventions significantly reduced the circulating concentrations of insulin compared to control mice fed with the HFHS diet, both when administered alone or in combination (*p* < 0.05 for all). Likewise, each supplement significantly reduced the MetS-induced increase in the HOMA-IR when administered separately (*p* < 0.05 each), although these values remained increased compared to control animals (*p* < 0.01). On the contrary, in mice supplemented with both CTE and BPL1^®^ HT, the reduction in HOMA-IR was more accentuated (*p* < 0.01), showing similar values to lean mice.

**Figure 1:**
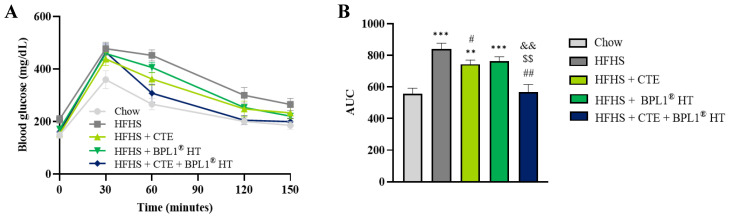
Glucose tolerance test (**A**) and area under the curve of the glucose tolerance test (**B**) of mice fed a high-fat diet/sucrose diet (HFHS), mice fed a high-fat diet/sucrose diet supplemented with Complex Tea Extract (HFHS + CTE), mice fed a high-fat diet/sucrose diet supplemented with BPL1^®^ HT (HFHS + BPL1TM HT), and mice fed a high-fat diet/sucrose diet supplemented with Complex Tea Extract and BPL1^®^ HT (HFHS + CTE + BPL1^®^ HT). Values are represented as mean value ± SEM; *n* = 8–10 mice/group. ** *p* < 0.01 vs. Chow; *** *p* < 0.001 vs. Chow; # *p* < 0.05 vs. HFHS; ## *p* < 0.01 vs. HFHS; $$ *p* < 0.01 vs. HFHS + CTE; && *p* < 0.01 vs. HFHS + BPL1^®^ HT.

The results from the glucose tolerance test (GTT) performed two weeks before the sacrifice of the animals (Figure 1) showed an increased area under the curve after (AUC) the intraperitoneal injection of a bolus of glucose in untreated mice with MetS compared to control mice, which indicates the presence of insulin resistance (*p* < 0.001). Supplementation with CTE, but not with BPL1^®^ HT, significantly reduced the AUC when administered alone (*p* < 0.05), thus improving insulin sensitivity. However, the AUC in this group (HFHS + CTE) was still significantly higher than the AUC from lean mice (*p* < 0.01). On the contrary, in mice co-administered with both supplements (CTE and BPL1^®^ HT), the AUC was comparable to that of control animals, indicating the reestablishment of insulin sensitivity.

### 2.6. Effect of CTE and BPL1^®^ HT Supplementation on the Activation of the PI3K/Akt Pathway and the Gene Expression of Inflammatory and Oxidative Stress Markers in Skeletal Muscle

**Figure 2:**
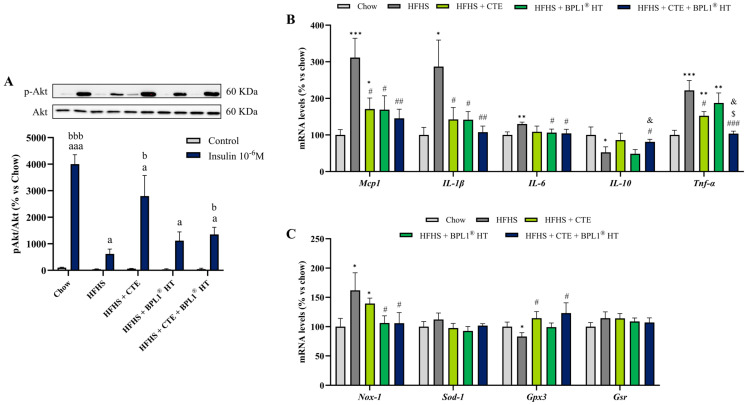
(**A**) p-Akt/Akt ratio in gastrocnemius explants after 15 min of incubation with or without 10^−6^ M insulin of mice fed a standard chow (Chow), mice fed a high-fat diet/sucrose diet (HFHS), mice fed a high-fat diet/sucrose diet supplemented with Complex Tea Extract (HFHS + CTE), mice fed a high-fat diet/sucrose diet supplemented with BPL1^®^ HT (HFHS + BPL1^®^ HT), and mice fed a high-fat diet/sucrose diet supplemented with Complex Tea Extract and BPL1^®^ HT (HFHS + CTE + BPL1^TM^ HT). mRNA levels of monocyte chemotactic protein-1 (Mcp-1), interleukin-1β (Il-1β), interleukin-6 (Il-6), interleukin-10 (Il-10), and tumor necrosis factor α (Tnf-α) (**B**), NADPH oxidase 1 (Nox-1), super oxide dismutase 1 (Sod-1), glutathione peroxidase 3 (Gpx-3), and glutathione reductase (Gsr) (**C**) in gastrocnemius of mice fed a standard chow (Chow), mice fed a high-fat diet/sucrose diet (HFHS), mice fed a high-fat diet/sucrose diet supplemented with Complex Tea Extract (HFHS + CTE), mice fed a high-fat diet/sucrose diet supplemented with BPL1^®^ HT (HFHS + BPL1^®^HT), and mice fed a high-fat diet/sucrose diet supplemented with Complex Tea Extract and BPL1^®^ HT (HFHS + CTE + BPL1^®^ HT). Values are represented as mean value ± SEM; *n* = 8–10 mice/group. a *p* < 0.05 vs. Control; aaa *p* < 0.001 vs. Control; b *p* < 0.05 vs. Insulin 10^−6^ M HFHS; bbb *p* < 0.001 vs. Insulin 10^−6^ M HFHS; * *p* < 0.05 vs. Chow; ** *p* < 0.01 vs. Chow; *** *p* < 0.001 vs. Chow; # *p* < 0.05 vs. HFHS; ## *p* < 0.01 vs. HFHS; ### *p* < 0.001 vs. HFHS; $ *p* < 0.05 vs. HFHS + CTE; & *p* < 0.05 vs. HFHS + BPL1^®^ HT.

Incubation with insulin for 15 min significantly increased the p-Akt/Akt ratio in gastrocnemius explants of mice from all experimental groups with this increase being statistically higher in explants of lean mice and mice with MetS supplemented with CTE either alone or in combination with BPL1^®^ HT (Figure 2A). The mRNA levels of inflammatory and oxidative stress markers in skeletal muscle are shown in Figure 2B and Figure 2C, respectively.

MetS was associated with an overexpression of the proinflammatory markers IL-1β (*p* < 0.05), IL-6 (*p* < 0.01), MCP-1 (*p* < 0.001), and TNF-α (*p* < 0.001) and with a downregulation in the gene expression of the anti-inflammatory cytokine IL-10 (*p* < 0.05). All of these alterations were prevented by the administration of CTE or BPL1^®^ HT either alone or in combination, except for the mRNA levels of IL-10 and TNF-α in mice supplemented only with BPL1^®^ HT. Interestingly, the supplementation with CTE and BPL1^®^ HT showed a synergistic effect on the gene expression of TNF-α since the mRNA levels of this cytokine were significantly lower in animals co-administered with both supplements compared to those administered only with one of them (*p* < 0.05).

Regarding oxidative stress markers, MetS was associated with an upregulation of the prooxidant enzyme NOX-1 that was prevented by BPL1^®^ HT, administered alone or in combination with CTE (*p* < 0.05 for both). On the other hand, supplementation with CTE, alone, or in combination with BPL1^®^ HT prevented the MetS-induced downregulation in the gene expression of the antioxidant enzyme GPX-3 (*p* < 0.05 for both).

### 2.7. Effect of CTE and BPL1^®^ HT Supplementation on the Activation of the PI3K/Akt Pathway in Liver Explants

**Figure 3:**
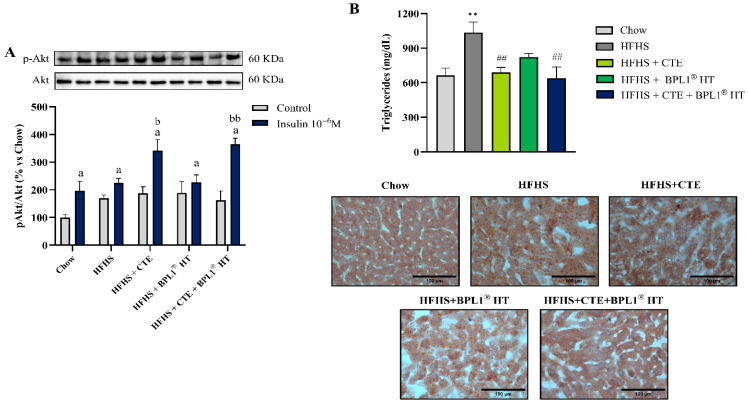
p-Akt/Akt ratio (**A**) in liver explants after 15 min of incubation with or without 10^−6^ M insulin of mice fed a standard chow (Chow), mice fed a high-fat diet/sucrose diet (HFHS), mice fed a high-fat diet/sucrose diet supplemented with Complex Tea Extract (HFHS + CTE), mice fed a high-fat diet/sucrose diet supplemented with BPL1^®^ HT (HFHS + BPL1^®^ HT), and mice fed a high-fat diet/sucrose diet supplemented with Complex Tea Extract and BPL1^®^HT (HFHS + CTE + BPL1^®^ HT). Quantification of triglycerides in the liver (**B**) and representative images of liver sections stained with oil red (lipid marker) of mice fed a standard chow (Chow), mice fed a high-fat diet/sucrose diet (HFHS), mice fed a high-fat diet/sucrose diet supplemented with Complex Tea Extract (HFHS + CTE), mice fed a high-fat diet/sucrose diet supplemented with BPL1^®^ HT (HFHS + BPL1^®^ HT), and mice fed a high-fat diet/sucrose diet supplemented with Complex Tea Extract and BPL1^®^ HT (HFHS + CTE + BPL1^®^ HT). The scale bar is equivalent to 100 µm. Values are represented as mean value ± SEM; *n* = 8–10 mice/group. a *p* < 0.05 vs. Control; b *p* < 0.05 vs. Insulin 10^−6^ M HFHS; bb *p* < 0.01 vs. Insulin 10^−6^ M HFHS; ** *p* < 0.01 vs. Chow; ## *p* < 0.01 vs. HFHS.

Incubation with insulin for 15 min significantly increased the p-Akt/Akt ratio in liver explants of mice from all experimental groups except in mice with MetS supplemented only with BPL1^®^ HT (Figure 3A). This activation was significantly higher in liver explants of mice with MetS supplemented with CTE either alone or in combination with BPL1^®^ HT (*p* < 0.05 and *p* < 0.01, respectively) compared to untreated MetS mice.

### 2.8. Effect of CTE and BPL1^®^ HT Supplementation on the Lipidic Content in the Liver

As shown in Figure 3B, MetS was associated with and increased triglyceride content in hepatic tissue (*p* < 0.01) that was prevented only by supplementation with CTE either alone or in combination with BPL1^®^ HT (*p* < 0.01).

### 2.9. Effect of CTE and BPL1^®^ HT Supplementation on the Gene Expression of Inflammatory and Oxidative Stress Markers in the Liver

**Figure 4:**
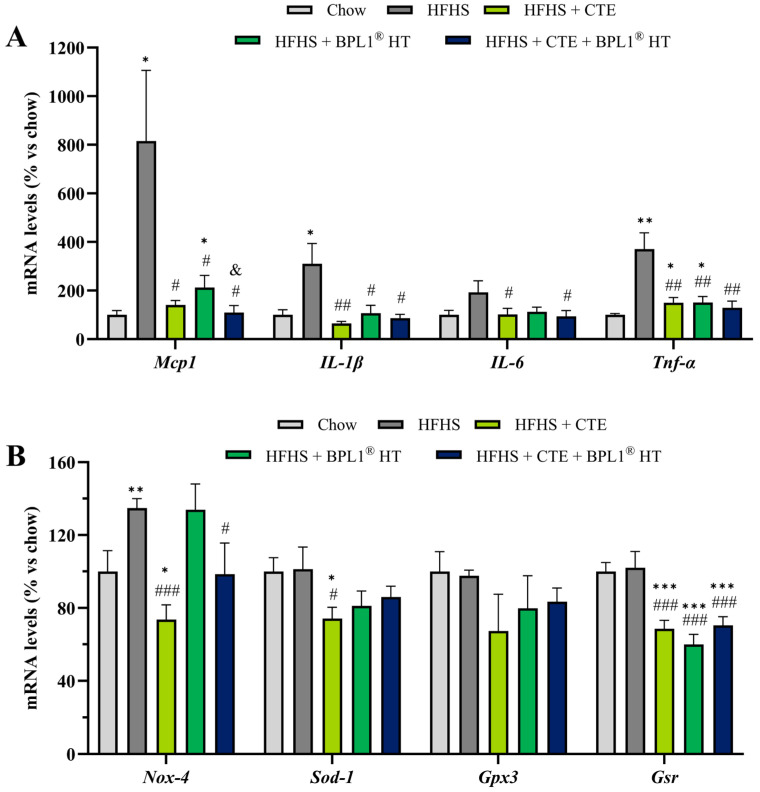
mRNA levels of monocyte chemotactic protein-1 (Mcp-1), interleukin-1β (Il-1β), interleukin-6 (Il-6), and tumor necrosis factor α (Tnf-α) (**A**), NADPH oxidase 4 (Nox-4), super oxide dismutase 1 (Sod-1), glutathione peroxidase 3 (Gpx-3), and glutathione reductase (Gsr) (**B**) in liver of mice fed a standard chow (Chow), mice fed a high-fat diet/sucrose diet (HFHS), mice fed a high-fat diet/sucrose diet supplemented with Complex Tea Extract (HFHS + CTE), mice fed a high-fat diet/sucrose diet supplemented with BPL1^®^HT (HFHS + BPL1^®^ HT), and mice fed a high-fat diet/sucrose diet supplemented with Complex Tea Extract and BPL1^®^HT (HFHS + CTE + BPL1^®^ HT). Values are represented as mean value ± SEM; *n* = 8–10 mice/group. * *p* < 0.05 vs. Chow; ** *p* < 0.01 vs. Chow; *** *p* < 0.001 vs. Chow; # *p* < 0.05 vs. HFHS; ## *p* < 0.01 vs. HFHS; ### *p* < 0.001 vs. HFHS; & *p* < 0.05 vs. HFHS + BPL1^®^ HT.

As shown in Figure 4A, MetS was associated with an upregulation in the mRNA levels of the proinflammatory markers TNF-α (*p* < 0.01), IL-1β (*p* < 0.05), IL-6 (*p* < 0.05), and MCP-1 (*p* < 0.05). The overexpression of these markers was prevented by the administration of CTE or BPL1^®^ HT either alone or in combination (*p* < 0.05). Interestingly, the supplementation with CTE and BPL1^®^ HT showed a synergistic effect on the gene expression of MCP-1 since the mRNA levels of this cytokine were significantly lower in animals co-administered with both supplements compared to those administered only with one of them (*p* < 0.05). Moreover, the separate administration of both products attenuated the MetS-induced overexpression of TNF-α, but this expression was still significantly higher than the one observed in control animals (*p* < 0.01). On the contrary, mice with MetS co-administered with both products showed similar hepatic TNF-α mRNA levels to those of healthy mice.

Regarding the gene expression of oxidative stress markers in the liver (Figure 4B), mice with MetS showed an upregulation of NOX-4 that was prevented by CTE supplementation either alone (*p* < 0.001) or in combination with BPL1^®^ HT (*p* < 0.05). In addition, supplementation with CTE alone significantly reduced the mRNA levels of SOD-1 compared to both control and non-supplemented mice with MetS (*p* < 0.05).

Furthermore, both treatments, CTE or BPL1^®^ HT administered alone or in combination, significantly reduced the gene expression of GSR (*p* < 0.001).

### 2.10. Effect of CTE and BPL1^®^ HT Supplementation on Adipocyte Size

**Figure 5:**
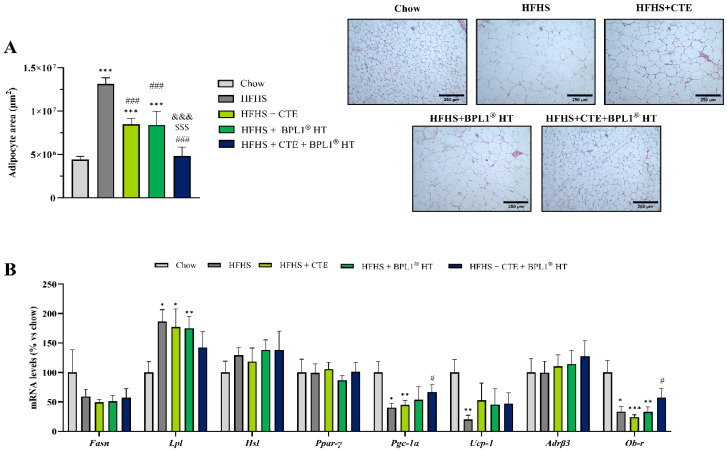
Size of adipocytes and representative images of H/E dying in sections of visceral adipose tissue (**A**). The scale bar is equivalent to 250 µm. mRNA levels of fatty acid synthase, lipoprotein lipase, hormone-sensitive lipase, peroxisome proliferator activated receptor, PPAR- coactivator-1, uncoupling protein-1, -3-adrenergic receptor, and leptin receptor (**B**) in retroperitoneal adipose tissue of mice fed a standard chow (Chow), mice fed a high-fat diet/sucrose diet (HFHS), mice fed a high-fat diet/sucrose diet supplemented with Complex Tea Extract (HFHS + CTE), mice fed a high-fat diet/sucrose diet supplemented with BPL1^®^ HT (HFHS + BPL1^®^ HT), and mice fed a high-fat diet/sucrose diet supplemented with Complex Tea Extract and BPL1^®^ HT (HFHS + CTE + BPL1^®^ HT). Values are represented as mean value ± SEM; *n* = 8–10 mice/group. * *p* < 0.05 vs. Chow; ** *p* < 0.01 vs. Chow; *** *p* < 0.001 vs. Chow; # *p* < 0.05 vs. HFHS; ### *p* < 0.001 vs. HFHS; $$$ *p* < 0.001 vs. HFHS + CTE; &&& *p* < 0.001 vs. HFHS + BPL1^®^ HT.

Mice fed with the HFHS diet showed a significant increase in the size of adipocytes in visceral adipose tissue compared to mice fed with chow (Figure 5A; *p* < 0.001). Supplementation with CTE or BPL1^®^ HT separately significantly decrease the adipocyte size (*p* < 0.001 for both), although mice from these groups already showed an increased adipocyte size compared to lean mice (*p* < 0.001 for both). However, mice from the HFHS + CTE + BPL1^®^ HT group showed decreased adipocyte size compared to mice from HFHS + CTE and HFHS + BPL1^®^ HT groups (*p* < 0.001 each) and similar adipocyte size to lean mice, indicating a complementary effect between them.

### 2.11. Effect of CTE and BPL1^®^ HT Supplementation on the Gene Expression of Markers Related to Lipid Metabolism

As shown in Figure 5B, MetS was associated with an overexpression of the lipogenic enzyme LPL (*p* < 0.05) which was not prevented either by CTE nor by BPL1^®^ HT when administered alone. Likewise, supplementation with just CTE or BPL1^®^ HT did not prevent the MetS-induced downregulation of PGC-1α (*p* < 0.05), UCP-1 (*p* < 0.01) and Ob-r (*p* < 0.05). Nevertheless, the combined administration of both supplements prevented the MetS-induced downregulation of PGC-1α and Ob-r (*p* < 0.05 for both).

### 2.12. Effect of CTE and BPL1^®^ HT Supplementation on the Gene Expression of Inflammatory and Oxidative Stress Markers in Visceral Adipose Tissue

**Figure 6:**
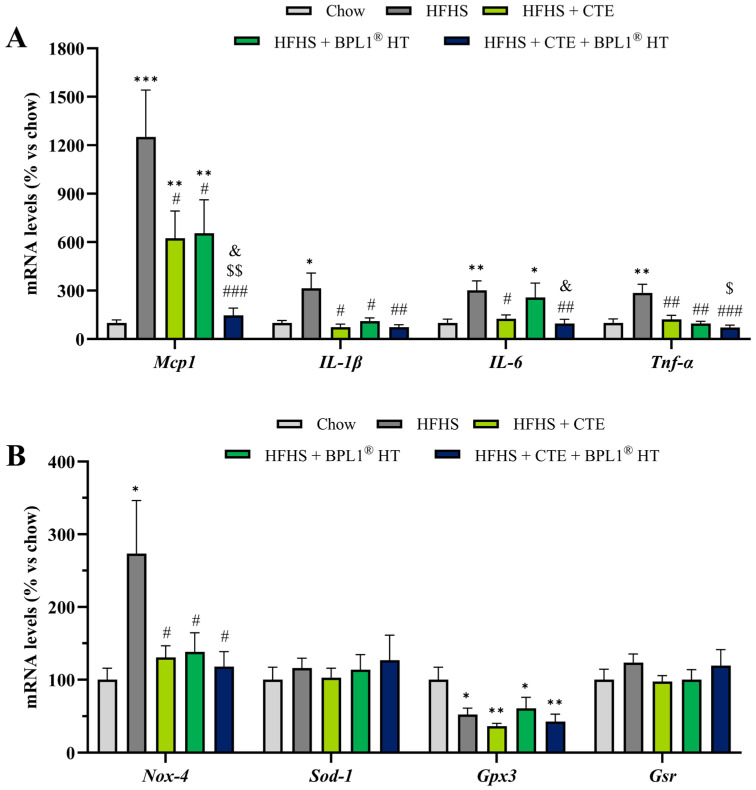
mRNA levels of monocyte chemotactic protein-1 (Mcp-1), interleukin-1β (Il-1β), interleukin-6 (Il-6), and tumor necrosis factor α (Tnf-α) (**A**), NADPH oxidase 4 (Nox-4), super oxide dismutase 1 (Sod-1), glutathione peroxidase 3 (Gpx-3), and glutathione reductase (Gsr) (**B**) in retroperitoneal adipose tissue of mice fed a standard chow (Chow), mice fed a high-fat diet/sucrose diet (HFHS), mice fed a high-fat diet/sucrose diet supplemented with Complex Tea Extract (HFHS + CTE), mice fed a high-fat diet/sucrose diet supplemented with BPL1^®^ HT (HFHS + BPL1^®^ HT), and mice fed a high-fat diet/sucrose diet supplemented with Complex Tea Extract and BPL1^®^ HT (HFHS + CTE + BPL1^®^ HT). Values are represented as mean value ± SEM; *n* = 8–10 mice/group. * *p* < 0.05 vs. Chow; ** *p* < 0.01 vs. Chow; *** *p* < 0.001 vs. Chow; # *p* < 0.05 vs. HFHS; ## *p* < 0.01 vs. HFHS; ### *p* < 0.001 vs. HFHS; $ *p* < 0.05 vs. HFHS + CTE; $$ *p* < 0.01 vs. HFHS + CTE; & *p* < 0.05 vs. HFHS + BPL1^®^ HT.

As shown in Figure 6A, mice with MetS showed an upregulation in the gene expression of the proinflammatory cytokines TNF-α (*p* < 0.01), IL-1β (*p* < 0.05), IL-6 (*p* < 0.01), and MCP-1 (*p* < 0.001). Supplementation with CTE prevented the MetS-induced overexpression of TNF-α (*p* < 0.01), IL-1β (*p* < 0.05) and IL-6 (*p* < 0.05) in visceral adipose tissue and significantly reduced the mRNA levels of MCP-1 (*p* < 0.05). However, the levels of this chemokine remained increased compared to healthy mice (*p* < 0.01). The same results were found after supplementation only with BPL1^®^ HT, except for the mRNA levels of IL-6, that were not reduced in the adipose tissue of mice supplemented only with the postbiotic. The combined administration of both supplements not only reversed the gene expression of TNF-α (*p* < 0.001), IL-1β (*p* < 0.01), and IL-6 (*p* < 0.01) to control levels, but also the gene expression of MCP-1, exerting an enhanced effect (*p* < 0.001).

The mRNA levels of the antioxidant enzymes SOD-1 and GSR were not modified either by MetS nor by supplementation with CTE or BPL1^®^ HT (Figure 5B). However, MetS was associated with an overexpression of NOX-4 and with a downregulation of GPX-3 (*p* < 0.05 for both). None of the ingredients prevented the MetS-induced downregulation of GPX3. However, both ingredients administered separately or in combination reversed the gene overexpression of NOX-4 to control levels (*p* < 0.05 for all).

### 2.13. Heart Rate, Diastolic Arterial Pressure, and Systolic Arterial Pressure

MetS was associated with an increased heart rate that was not attenuated by any of the ingredients either alone or in combination, as shown in Table 4. However, both supplements administered separately or in combination prevented the MetS-induced increase in systolic blood pressure, with this effect being more evident in mice supplemented with CTE (*p* < 0.001) than in mice supplemented with BPL1^®^ HT (*p* < 0.01). Diastolic blood pressure did not differ among experimental groups.

### 2.14. Vascular Response of Aortic Rings to the Vasoconstrictors ET-1 and AngII and to NA in the Presence/Absence of L-NAME

**Figure 7:**
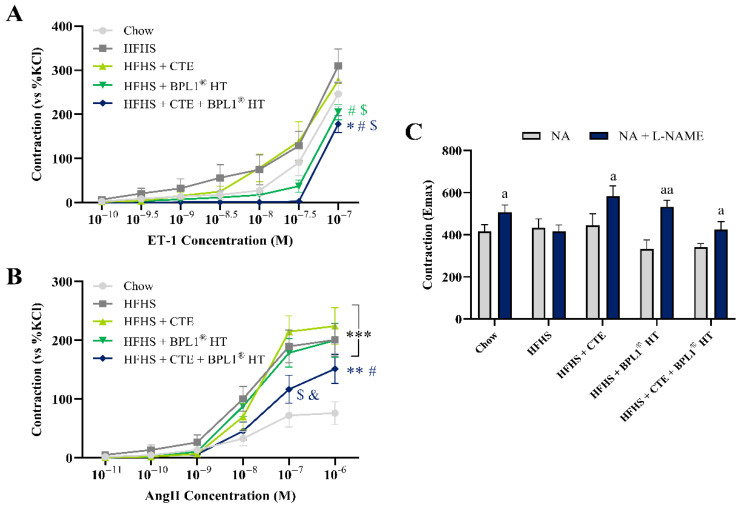
Contraction of the abdominal aortic rings to endothelin-1 (ET-1) (10^−10^–10^−7^ M) (**A**), contraction to angiotensin-II (AngII) (10^−11^–10^−6^ M) (**B**), and Emax contraction to noradrenaline (NA) (10^−9^–10^−4^ M) in the presence/absence of N-Nitro-L-arginine methyl ester (L-NAME) (10^−4^ M) (NA/NA + L-name) (**C**) of mice fed a standard chow (Chow), mice fed a high-fat diet/sucrose diet (HFHS), mice fed a high-fat diet/sucrose diet supplemented with Complex Tea Extract (HFHS + CTE), mice fed a high-fat diet/sucrose diet supplemented with BPL1^®^ HT (HFHS + BPL1^®^ HT), and mice fed a high-fat diet/sucrose diet supplemented with Complex Tea Extract and BPL1^®^ HT (HFHS + CTE + BPL1^®^ HT). Values are represented as mean value ± SEM; *n* = 8–10 mice/group. * *p* < 0.05 vs. Chow; ** *p* < 0.01 vs. Chow; *** *p* < 0.001 vs. Chow; # *p* < 0.05 vs. HFHS; $ *p* < 0.05 vs. HFHS + CTE; & *p* < 0.05 vs. HFHS + BPL1^®^ HT; a *p* < 0.05 vs. NA of its corresponding experimental group; aa *p* < 0.01 vs. NA of its corresponding experimental group.

MetS did not modify the vascular response of aorta segments to ET-1 (Figure 7A). On the contrary, aortic rings from non-supplemented mice with MetS showed an increased response to AngII (Figure 7B; *p* < 0.001). Supplementation with CTE alone did not affect the vascular response of aorta segments either to ET-1 or Ang-II, whereas supplementation just with BPL1^®^ HT significantly decreased the response to ET-1 (*p* < 0.05). Moreover, the combination of both supplements attenuated the MetS-induced increased response to AngII (*p* < 0.05).

No differences were found among experimental groups in the vascular response of aortic rings to NA in basal conditions (Figure 7C). However, in the presence of L-NAME, there was an increased response to NA in all experimental groups (*p* < 0.05 for all), except in aorta segments from untreated mice with MetS.

### 2.15. Assessment of the Endothelium-Dependent and Endothelium-Independent Relaxation in Aorta Segments

**Figure 8:**
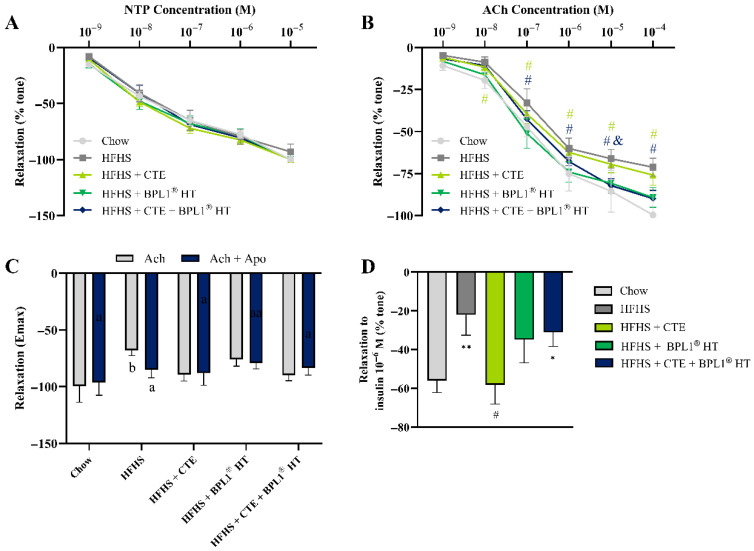
The relaxation of thoracic aortic segments to sodium nitroprusside (NTP) (10^−9^–10^−5^ M) (**A**), relaxation to acetylcholine (ACh) (10^−9^–10^−4^ M) (**B**), Emax relaxation to acetylcholine (10^−9^–10^−4^ M) in the presence/absence of apocynin (ACh/ACh + Apo) (10^−6^ M) (**C**), and relaxation to insulin 10^−6^ M dose (**D**) of mice fed a standard chow (Chow), mice fed a high-fat diet/sucrose diet (HFHS), mice fed a high-fat diet/sucrose diet supplemented with Complex Tea Extract (HFHS + CTE), mice fed a high-fat diet/sucrose diet supplemented with BPL1^®^ HT (HFHS + BPL1^®^ HT), and mice fed a high-fat diet/sucrose diet supplemented with Complex Tea Extract and BPL1^®^ HT (HFHS + CTE + BPL1^®^ HT). Values are represented as mean value ± SEM; *n* = 8–10 mice/group. * *p* < 0.05 vs. Chow; ** *p* < 0.01 vs. Chow; # *p* < 0.05 vs. HFHS; & *p* < 0.05 vs. HFHS + BPL1^®^ HT; a *p* < 0.05 vs. Ach of its corresponding experimental group; b *p* < 0.05 vs. Ach of Chow group.

**Figure 9:**
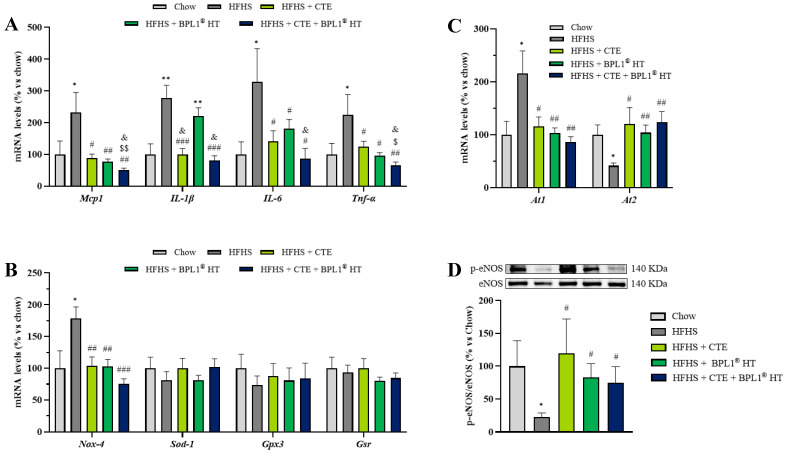
mRNA levels of monocyte chemotactic protein-1 (Mcp-1), interleukin-1β (Il-1β), interleukin-6 (Il-6), and tumor necrosis factor α (Tnf-α) (**A**), NADPH oxidase 4 (Nox-4), super oxide dismutase 1 (Sod-1), glutathione peroxidase 3 (Gpx-3) and glutathione reductase (Gsr) (**B**), and angiotensin II receptor type 1 (At1) and angiotensin II receptor type 2 (At2) (**C**), and p-eNOS/eNOS ratio (**D**) in the aorta of mice fed a standard chow (Chow), mice fed a high-fat diet/sucrose diet (HFHS), mice fed a high-fat diet/sucrose diet supplemented with Complex Tea Extract (HFHS + CTE), mice fed a high-fat diet/sucrose diet supplemented with BPL1^®^ HT (HFHS+ BPL1^®^ HT), and mice fed a high-fat diet/sucrose diet supplemented with Complex Tea Extract and BPL1^®^ HT (HFHS + CTE + BPL1^®^ HT). Values are represented as mean value ± SEM; *n* = 8–10 mice/group. * *p* < 0.05 vs. Chow; ** *p* < 0.01 vs. Chow; # *p* < 0.05 vs. HFHS; ## *p* < 0.01 vs. HFHS; ### *p* < 0.001 vs. HFHS; $ *p* < 0.05 vs. HFHS + CTE; $$ *p* < 0.01 vs. HFHS + CTE; & *p* < 0.05 vs. HFHS+ BPL1^®^ HT.

No significant changes were found among experimental groups in the endothelium-independent relaxation in response to NTP (Figure 8A). On the contrary, MetS was associated with a significant reduction in the endothelium-dependent relaxation measured as the vascular response of aortic rings to accumulative concentrations of Ach (*p* < 0.05; Figure 8B) or the vascular response of aorta segments to a single dose of insulin (*p* < 0.01; Figure 8D), indicating the presence of endothelial dysfunction. CTE supplementation, alone or in combination with BPL1^®^ HT, prevented MetS-induced endothelial dysfunction (*p* < 0.05), whereas supplementation with BPL1^®^ HT alone did not have any impact either in the vascular response to Ach or the vascular response to insulin. The altered endothelial function induced by MetS was associated with a significant downregulation in the expression of the activated endothelial nitric oxide synthase in arterial tissue (*p* < 0.05; Figure 9D) and with a significant decrease (*p* < 0.05) in the release of nitric oxide (NO) that were prevented by the ingredients’ supplementation (Table 5).

### 2.16. Effect of CTE and BPL1^®^ HT Supplementation on the Gene Expression of Inflammatory and Oxidative Stress Markers in Aortic Tissue

As shown in Figure 9A, mice with MetS showed an upregulation in the gene expression of the proinflammatory cytokines TNF-α (*p* < 0.05), IL-1β (*p* < 0.01), IL-6 (*p* < 0.05), and MCP-1 (*p* < 0.05) in the aorta. Supplementation with CTE or BPL1^®^ HT, both separately or in combination, prevented the MetS-induced overexpression of all proinflammatory markers, except for the gene expression of IL-1β, which was only significantly reduced by CTE, both alone or in combination with BPL1^®^ HT (*p* < 0.001 for both). In addition, the reduction in the mRNA levels of TNF-α was more accentuated in the aortic tissue of mice co-administered with the combination of both supplements (*p* < 0.01) compared to those supplemented only with one of them (*p* < 0.05), pointing to a synergistic effect between them.

In aortic tissue, none of the oxidative stress markers were modified either by MetS or by supplementation with CTE or BPL1^®^ HT except for the mRNA levels of NOX-4 that were significantly increased in mice fed with the HFHS diet and normalized in mice supplemented with CTE or BPL1^®^ HT both alone (*p* < 0.01) or in combination (*p* < 0.001) (Figure 9B). Furthermore, all ingredients prevented the MetS-induced alterations in the gene expression of the AngII receptors At1 and At2 (Figure 9C).

### 2.17. Modulation of the Gut Microbiota by CTE and BPL1^®^ HT Supplementation

A total of 909 ASVs were annotated as bacteria and passed the prevalence filter. In total, 12% of these ASV were classified at genera level. To evaluate if CTE, BPL1^®^ HT or a combination of both can modulate gut microbiota in a context of HFHS diet, bacterial α-diversity, differential abundance of bacteria, and association between clinical/molecular and microbial features were assessed.

**Figure 10:**
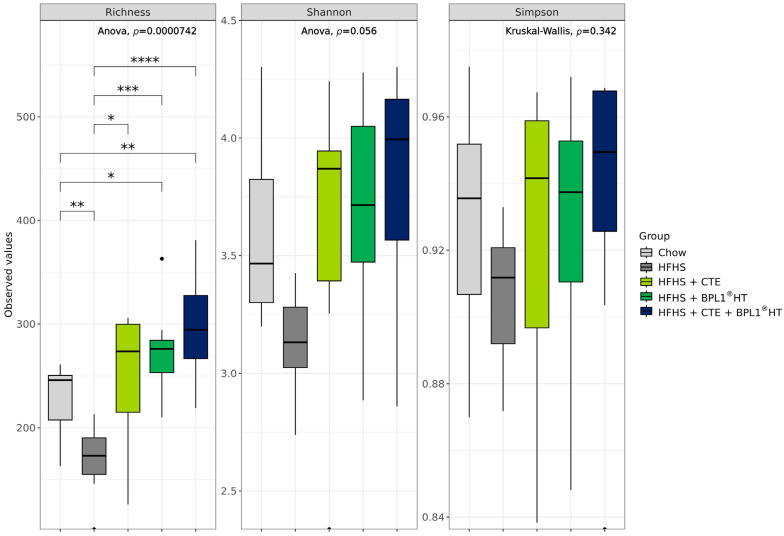
Boxplots of alpha diversity (richness, Shannon, and Simpson indexes) of mice fed a standard chow (Chow), mice fed a high-fat diet/sucrose diet (HFHS), mice fed a high-fat diet/sucrose diet supplemented with Complex Tea Extract (HFHS + CTE), mice fed a high-fat diet/sucrose diet supplemented with BPL1^®^ HT (HFHS + BPL1^®^ HT), and mice fed a high-fat diet/sucrose diet supplemented with Complex Tea Extract and BPL1^®^ HT (HFHS + CTE + BPL1^®^ HT) for 23 weeks. An analysis of variance (ANOVA) or a Kruskal–Wallis test was performed for parametric or non-parametric data. Student’s *t*-test was performed as ANOVA post hoc analysis. * *p* < 0.05, ** *p* < 0.01, *** *p* < 0.001, **** *p* < 0.0001 between groups.

Regarding alpha diversity, three metrics were calculated. Mice in HFHS showed a decrease in bacterial richness compared to mice in Chow diet (Figure 10, *p* < 0.05). Supplementation of CTE or BPL1^®^ HT prevented richness reduction, and the combination of both ingredients not only prevented the loss of richness by HFHS, but also increased richness compared to mice in Chow diet. The same pattern was observed in Shannon (Figure 10) and Simpson (Figure 10) without reaching statistical significance. Mice supplemented with a blend of CTE + BPL1^®^ HT showed a tendency of higher indexes of alpha diversity compared to mice in CTE or BPL1^®^ HT alone (Figure 10).

**Figure 11:**
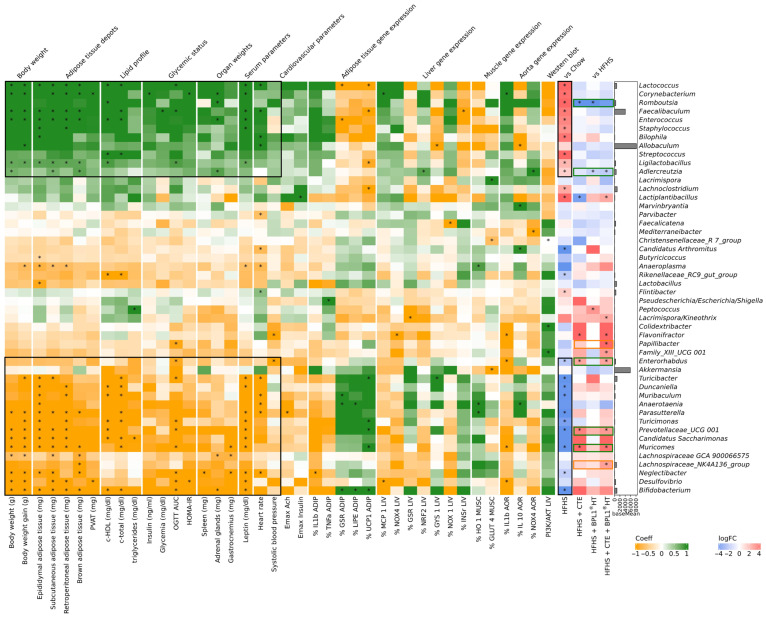
Association heatmaps showing the Maaslin2 Coefficient (Coeff) between clinical variables, gene expression and protein expression, and gut genera abundances. Left heatmap in green and orange: green color means the feature is directly associated with taxa abundance, while orange color is inversely associated. Right heatmap in red and blue: red means that the taxon is over-represented in the first group of the comparison, while blue means that the taxon is under-represented in the first group. Black boxes highlight taxa associated with several clinical parameters of metabolic syndrome and altered by high-fat diet/sucrose diet (HFHS) compared to standard chow (Chow); green boxes highlight the taxa that were altered by HFHS compared to Chow and modulated by the supplementation with Complex Tea Extract (HFHS + CTE), BPL1^®^ HT (HFHS+ BPL1^®^ HT) or the blend Complex Tea Extract and BPL1^®^ HT (HFHS + CTE + BPL1^®^ HT). Orange boxes highlight the taxa that was modulated only by the blend, and that are associated negatively with clinical parameters of metabolic syndrome. * adj. *p* < 0.05 in left heatmap means the feature is significantly associated with taxa abundance. * adj. *p* < 0.05 in right heatmap means the differential abundance between groups and the taxon is present in at least 50% of samples of one of the compared groups. BaseMean bar plots show the mean abundances of each genera.

Multivariate analysis using MaAsLin2 identified several genera with significant correlation with clinical parameters in HFHS associated with MetS condition. Specifically, 10 bacterial genera (*Lactococcus*, *Corynebacterium*, *Romboutsia*, *Faecalibaculum*, *Enterococcus*, *Staphylococcus*, *Bilophia*, *Streptococcus*, *Ligilactobacillus*, *Adlercreutzia*), whose abundance were increased by HFHS diet, showed a positive association with clinical parameters (Figure 11, black boxes at the top). Conversely, the abundance of another 10 genera (*Bifidobacterium*, *Negletibacter*, *Muricomes*, *Prevotellaceae UCG 001*, *Turicimonas*, *Parasutterella*, *Anaerotaenia*, *Muribaculum*, *Duncaniella*, *Turicibacter*, *Enterohabdus*), whose abundance were decreased by HFHS diet, was negatively correlated with these parameters (Figure 11, black boxes at the bottom).

Supplementation with CTE, BPL1^®^ HT, or CTE + BPL1^®^ HT modulated the abundance of 5 of these 20 genera (Figure 11, taxa in green boxes).

Specifically, diet supplemented with CTE or CTE + BPL1^®^ HT increased the abundance of *Muricomes*, *Prevotellaceae* UCG 001, and *Enterorhabdus* compared to mice in HFHS alone, reaching the same values as those of mice fed with chow in the case of *Muricomes* and *Enterorhabdus* (Supplementary Figure S1).

For one side, *Bifidobacterium*, negatively associated with several clinical parameters, decreased with HFHS diet, while the ingredients increased it, showing the blend the highest increase. On the other side, supplementation with TC or BPL1^®^ HT reduced the abundance of *Romboutsia* and BPL1^®^ HT and CTE + BPL1^®^ HT reduced *Adlercreutzia*, genera positively associated with MetS parameters and increased in HFHS diet (Supplementary Figure S1).

Additionally, only the supplementation of CTE + BPL1^®^ HT increased the abundance of *Lachnospiraceae*_NK4A136_group (associated negatively with brown adipose tissue) and *Papilibacter* (associated negatively with OGTT AUC) (Supplementary Figure S2).

At ASVs level, the same pattern was observed: compared to Chow mice, the HFHS diet increased the abundance of 14 ASVs positively associated with MetS parameters and reduced 18 ASVs negatively associated (Supplementary Figure S3). From negatively associated ASVs, CTE and CTE + BPL1^®^ HT were able to restore the abundance comparable to Chow mice of ASV126 *Enterorhabdus* (that could be annotated up to genus level) and only CTE + BPL1^®^ HT restored ASV78 *Oscillospiraceae* (that could be annotated up to family level) (Supplementary Figure S4). CTE and CTE + BPL1^®^ HT prevented the severe decrease in ASV16 *Bifidobacterium pseudolongum*, a similar trend as in genera (Supplementary Figure S4). Moreover, the ASVs positively associated with MetS, only the blend completely prevented the increase in ASV83 *Flintibacter butyricus* and CTE prevented the increase in ASV95 *Romboutsia timonensis* caused by HFHS diet (Supplementary Figure S4).

## 3. Discussion

The present study demonstrates that the combined administration of a standardized green and black tea extract (ADM Tea Complex, CTE) and the heat-treated postbiotic strain BPL1^®^ HT exerts complementary effects in ameliorating cardiometabolic alterations associated with metabolic syndrome (MetS) in a murine model. Compared to single-ingredient supplementation, the co-administration normalized body weight, adiposity, visceral adipocyte hypertrophy, dyslipidemia, insulin resistance, hepatic steatosis, vascular dysfunction, and inflammatory/oxidative markers to levels comparable to healthy controls. These findings highlight the potential of combining polyphenol-rich botanicals with microbiota-targeted interventions for MetS management.

The restoration of glucose homeostasis and insulin sensitivity by CTE + BPL1^®^ HT co-treatment is linked to the reactivation of PI3K/Akt signaling in metabolic tissues. In liver and skeletal muscle, the combination restored insulin-stimulated Akt phosphorylation, a critical node in insulin signaling pathways that regulate GLUT4 translocation and glucose uptake [4]. This aligns with a previous study reported by our group in which supplementation with the same tea extract prevented MetS-induced insulin resistance through the activation of the PI3K/Akt pathway in the main metabolic tissues such as the liver, the skeletal muscle, and the adipose tissue [29]. The beneficial effects of tea on glucose metabolism are described in the literature and are due, at least in part, to the presence of tea polyphenols like (-)-epigallocatechin-3-gallate (EGCG), which enhances insulin sensitivity by reducing IRS-1 serine phosphorylation and promoting Akt activation [34] and by activating the AMPK pathway [35]. However, in our study, none of the supplements activated the AMPK pathway, which indicates that the beneficial metabolic effects are mainly due to the activation of the PI3K/Akt pathway.

On the other hand, BPL1^®^ HT has also been reported to exert insulin sensitizing effects both in experimental animals [32] and in humans [36] due to its antiadipogenic [37] and microbiome modulating effects [36].

The synergy between both supplements on insulin sensitivity most likely arises from complementary targeting of insulin resistance and reduction in adiposity, since both enhance PI3K/Akt activation in the liver and skeletal muscle and mitigate adiposity and adipose tissue inflammation.

In addition to the antiadipogenic and insulin sensitizing effects, another important finding of our study is that the administration of both products, separately or in combination, prevents the development of hypertension, with this effect being more evident in those animals supplemented with CTE. The antihypertensive effects of CTE have already been reported by our group not only in the context of MetS [29] but also in an experimental model of hypertension induced by AngII infusion [31]. Likewise, other tea extracts, especially green and black tea, are also reported to lower arterial blood pressure both in experimental animals [38,39,40,41] and in humans [40,41] due to the presence of bioactive substances, mostly flavan-3-ols [42,43]. Likewise, supplementation only with BPL1^®^ HT has also been reported to exert antihypertensive effects in an experimental model of hypertension induced by AngII infusion [33]. In the present study, the positive effects of BPL1^®^ HT lowering blood pressure were associated with a partial increase in eNOS activation and with a decreased arterial contraction in response to the vasoconstrictor peptide ET-1, whereas the antihypertensive effects of CTE were basically due to an improvement in endothelial function. These results agree with previous studies in which tea enhances eNOS coupling and NO production via PI3K/Akt-dependent pathways due, at least in part, to the presence of tea polyphenols, mostly flavan-3-ols such as EGCG, EC, etc. [44,45]. However, despite the partial increase in eNOS activation, in this study, BPL1^®^ HT supplementation did not improve the endothelium-dependent relaxation nor the release of NO by aorta segments, which disagrees with a previous study in which the antihypertensive effects of BPL1^®^ HT were the result of improved endothelial function [33]. These discrepancies might be due to differences in the experimental models (AngII-induced hypertension vs. MetS), the age of the animals, or the duration of the treatments.

Importantly, the combined supplementation of CTE and BPL1^®^ HT not only decreased the vascular response of aortic segments to ET-1 and restored endothelial function to control levels but also reduced the vascular response of aortic rings to AngII, an effect that was not present when the supplements were administered separately. The co-administration’s superior efficacy against angiotensin II responses suggests synergistic inhibition of RAAS hyperactivity, a key driver of hypertension in MetS. The decreased vasoconstrictor activity of AngII in response to tea components has already been reported both in vitro [46,47] and in vivo [48] and seems to be the result of the direct inhibitory effect of tea polyphenols on angiotensin converting enzyme [49]. Moreover, supplementation with CTE is reported to stimulate angiotensin converting enzyme 2 (ACE2), which increases the circulating levels of the vasodilating peptide Ang 1–7 [31], thus contributing to the reduction in blood pressure. In addition to the synergic effect on the vasoconstrictor response to AngII, another important finding of our work is that the co-administration with both supplements also exerted synergistic effects on the gene expression of oxidative stress and inflammation markers in metabolic and cardiovascular tissues, with this fact arising as a possible mechanism for their enhanced beneficial cardiometabolic effects. This is critical given the bidirectional relationship between oxidative stress and inflammation in MetS pathogenesis: ROS activate NF-κB and NLRP3 inflammasomes, driving cytokine production that further exacerbates insulin resistance and endothelial dysfunction [16]. The synergic effect between CTE and BPL1^®^ HT is especially evident in the gene expression of several proinflammatory cytokines such as IL-6, TNF-α, and MCP-1 in visceral adipose tissue and in arterial tissue, pointing to the anti-inflammatory effect as the main mechanism mediating the beneficial effects of the blend in these tissues. The positive effects of both supplements administered alone or in combination reducing the mRNA levels of several markers related to inflammation and oxidative stress agree with previous studies from our group in which the anti-inflammatory and antioxidant effects of both CTE and BPL1^®^ HT were reported in animal models of MetS [29,30] and AngII-induced hypertension [31,33]. Likewise, there are several studies reporting the antioxidant and anti-inflammatory effects of other tea extracts in a context of metabolic syndrome [50,51,52]. However, although several postbiotics are reported to exert antioxidant and anti-inflammatory effects in different pathological conditions such as colitis [53,54], this is the first study reporting the anti-inflammatory and antioxidant effects of a postbiotic in an experimental model of MetS.

In addition, the synergistic effects between the two ingredients may be related, at least in part, to the effects of both supplements modulating the gut microbiome. In this regard, it is reported that green tea polyphenols, mainly catechins like EGCG, EGC, ECg, EC, etc., reach the colon where gut microbes metabolize them into smaller bioactive compounds. These metabolites can modulate microbial composition by stimulating beneficial bacteria (e.g., *Bifidobacterium* spp., *Akkermansia* spp.) and suppressing harmful species (e.g., *Enterobacteriaceae*, *Fusobacterium*) [55]. Likewise, the beneficial effects of BPL1^®^ HT are reported to be mediated through an increase in gut *Akkermansia* [36]. Taking this into account, additional experiments were performed to investigate whether supplementation with the ingredients exerts any effects on modulating the gut microbiome.

Our results show that both CTE and BPL1^®^ HT, alone and in combination, prevented the reduction in bacterial richness induced by HFHS diet. However, only mice supplemented with the blend reached levels of bacterial richness higher than mice fed with chow diet, highlighting the synergist effect of CTE and BPL1^®^ HT. The lack of differences between the blend and the individual ingredients could be explained by the tendency of both CTE and BPL1^®^ HT to increase bacterial richness compared to chow. High-calorie diets can cause gut dysbiosis, leading to reduced microbial diversity, greater energy extraction, impaired gut barrier function, and chronic low-grade inflammation. This triggers metabolic endotoxemia, oxidative stress, and abnormal regulation of genes controlling lipid and glucose metabolism as well as inflammatory pathways, all of which contribute significantly to metabolic disorders (Dabke 2019). In fact, a study in a large human population found that higher alpha diversity was associated with lower insulin resistance and lower prevalence of type 2 diabetes [56].

Association and differential abundance analysis revealed that CTE, BPL1^®^ HT, and the combination of both were able to modulate the abundance of bacterial biomarkers associated with clinical parameters of MetS. In this regard, the blend showed a tendency to increase the abundance of beneficial bacteria such as *Bifidobacterium* compared to CTE or BPL1^®^ HT alone. The synergist effect of CTE and BPL1^®^ HT was clearer at ASV level where mice supplemented with the blend had significantly lower abundance of *Flintibacter butyricus*, higher abundance of ASV78 *Oscillospiraceae*, and a tendency of higher *Bifidobacterium pseudolongum* compared to ingredients alone. *Bifidobacterium* are commensal bacteria used as probiotics, with positive reports on improving metabolic diseases [57]. Prebiotics such as mannan-oligosaccharides and compounds such as glycerol monolaurate have been reported to improve obesity and metabolic disorders which were associated with the increased abundance of *B. pseudolongum* [58,59]. The blend modulates other *Bifidobacterium* species besides *Bifidobacterium animalis sub. lactis*, the strain from BPL1^®^ HT derived from. Regarding *F. butyricus*, a bacterium that in our study correlated positively with several MetS clinical parameters, only the blend completely prevented the increase in this bacterium produced by HFHS. This is in line with the other study where it was observed that *F. butyricus* increase during lard- and bile acid-fed mice [59].

The clearer tendency of synergies between CTE and BPL1^®^ HT was observed on the alpha diversity analysis, which is performed at ASV level, independent of the level of taxonomic annotation. Only 12% of the identified ASV could be annotated to genera level, with this fact possibly limiting the biomarker analysis (clinical associations and differential abundance).

In conclusion, the combined supplementation of standardized green and black tea extracts CTE and the postbiotic BPL1^®^ HT demonstrated synergistic effects in ameliorating a wide range of cardiometabolic alterations associated with MetS in a murine model. Co-administration improved parameters such as body weight, adipocyte hypertrophy, insulin sensitivity, lipid profiles, hepatic steatosis, vascular function, and the expression of inflammation and oxidative stress, achieving effects not observed with each supplement alone. Moreover, this study demonstrates that CTE, BPL1^®^ HT, and the combination of both modulates gut microbiota in the context of HFHS, highlighting the capacity of the blend to increase bacterial richness and beneficial bacteria like *Bifidobacterium* species, while preventing the increase in potential opportunistic bacteria like *F. butyricus*. The beneficial effects on gut microbiota effect, together with the positive effects of the blend on clinical parameters, suggest that the combined supplementation with CTE and BPL1^®^ HT could be a novel intervention for alleviating the metabolic and cardiovascular alterations associated with MetS.

Despite these promising findings, results of this study were obtained in a specific animal model; therefore, extrapolation to human physiology requires caution and should be validated in clinical trials.

## 4. Materials and Methods

### 4.1. Reagents and Chemicals

For the liquid chromatography, methylxanthines (caffeine, theobromine and theophylline), monomeric flavan-3-ols [(+)-catechin, catechin gallate, (-)-Epicatechin (EC), Epicatechin-3-gallate (ECg), epigallocatechin (EGC), epigallocatechin-3-gallate (EGCg), green tea catechin mix], theaflavins [theaflavin, theaflavins mix (tea extract from Camellia sinensis)], and gallic acid, were purchased from Merck (Madrid, Spain) and Phytolab (Vestenbergsgreuth, Germany). These standards were used for the identification and/or quantification of characteristic major bioactive components from Complex Tea Extract and their digested fractions. Trifluoroacetic acid, acetonitrile, methanol, and water of chromatographic quality solvents were purchased from VWR (Valencia, Spain).

For in vitro digestion, phosphate buffer saline (PBS) pH 7.4, pepsin from porcine gastric mucosa, α-amylase, pancreatin enzymes, bile powder, hydrogen chloride acid (HCl), and Bicarbonate (NaHCO_3_) products were purchased from Merck (Madrid, Spain).

### 4.2. Commercial Tea Extract

The industrial-scale product, Complex Tea Extract or Tea Complex (*Camellia sinensis* L.), standardized to contain total flavan-3-ols (both monomeric forms and theaflavins) as well as methylxanthines (caffeine, theobromine, and theophylline), was obtained directly through a water-extraction process applied to a proprietary blend of green and black tea leaves, as previously described [29]. This extract was provided by ADM^®^.

### 4.3. High-Performance Liquid Chromatography (HPLC)

Flavan-3-ols, methylxanthines, theaflavins, and gallic acid were analyzed according to the method used by De la Fuente-Muños et al. [29]. The HPLC system used for the analysis consisted of a Shimadzu Nexera XR UHPLC (70 MPa) coupled to a photodiode array detector (SPD-M40 model) from Izasa Scientific (Madrid, Spain). Chromatographic separation was performed using a Zorbax Eclipse Plus C18 octadecyl silane column (250 mm × 4.6 mm, 5 µm) along with its corresponding precolumn (Agilent Technologies, Barcelona, Spain).

Detection was conducted at 275 nm, with the oven temperature set to 32 °C. The flow rate was maintained at 1.0 mL/min, and the injection volume was 2 µL. A binary gradient system was employed for the chromatographic separation, consisting of Phase A (5% *v*/*v* acetonitrile and 0.035% *v*/*v* trifluoroacetic acid) and Phase B (50% *v*/*v* acetonitrile and 0.025% *v*/*v* trifluoroacetic acid). The initial gradient conditions were set to A:B (90:10), which gradually increased to 20% B at 10 min, followed by 40% B between 25 and 27 min. Finally, the column was reequilibrated to the initial gradient conditions for 3 min before the next injection.

Identification of monomeric and oligomeric flavan-3-ols (gallocatechin, epigallo-catechin, catechin, epicatechin, epigallocatechin-3-gallate, gallocatechin-3-gallate, epi-catechin-3-gallate, and catechin-3-gallate), theaflavins or oligomeric flavan-3-ols (theaflavin, theaflavin-3-monogallate, theaflavin-3′-monogallate, and theaflavin-3,3′-digallate), methylxanthines (theobromine, theophylline, and caffeine), and gallic acid was performed by comparing retention times, UV-Vis spectra, and corresponding reference standards. Quantification was carried out using an external calibration curve with at least five different concentration points (r^2^ = 0.99). The results were expressed as percentages (%, dry basis). The sum of monomeric flavan-3-ols, methylxanthines, and theaflavins was quantified as epigallocatechin gallate (EGCg), caffeine, and theaflavin equivalents, respectively.

### 4.4. In Vitro Digestion

Static in vitro digestion was performed according to the method described by Hollebeeck et al. [60], structured into three stages, oral, gastric, and intestinal digestion, while taking into account the nutritional composition of the Tea Complex (Table 1). Briefly, 2 g of Tea Complex was dissolved in 20 mL of PBS (final volume of approximately 20–30 mL). The oral digestion phase lasted 5–10 min under continuous shaking (model Grant bio PSU-10i; VWR, Spain) at pH 6.8–6.9 and 37 °C under aerobic conditions, using 3.9 units of α-amylase/mL dissolved in PBS in 50 mL centrifuge tubes.

Subsequently, the pH of the medium was adjusted to 2–3 (at 37 °C) for the gastric digestion phase, which was carried out with 71.2 units of pepsin/mL dissolved in 0.1 M HCl under anaerobic conditions. This phase was conducted under continuous slow shaking for 90–120 min to mimic gastric digestion, with a final volume of 40–50 mL.

Finally, the intestinal (duodenal) phase was conducted for 150 min at pH 7.0–7.4 and 37 °C, using 9.2 mg of pancreatin/mL and 55.2 mg of bile extract/mL (in a 1:6 enzyme-to-bile ratio) under anaerobic conditions, with a final volume of approximately 80–90 mL. To stop the reaction at the end of each phase, samples were heated to 90 °C and then centrifuged at 7000× *g* (Eppendorf 5804R; VWR, Spain) for 10 min at 4 °C. The resulting supernatants were dried in a vacuum oven (Vaciotem-T Selecta, Spain) and stored until further analysis.

### 4.5. Animals

Sixteen-week-old C57/BL6J mice were housed in pairs and maintained in climate-controlled quarters under controlled conditions of humidity (50–60%) and temperature (22–24 °C), and with a 12 h light cycle. Experiments were performed with the approval of the Ethical Committee of the Community of Madrid (Madrid, Spain) (PROEX 286.2-22).

Mice were fed ad libitum and divided into five experimental groups (*n* = 8–9): mice fed with a standard chow (Chow/Lean/Controls); mice fed a high-fat/high-sucrose diet containing 58% kcal from fat with sucrose (HFHS) and no supplemented with any ingredient (HFHS/untreated mice with MetS), mice fed a high-fat/high-sucrose (HFHS) diet containing 58% kcal from fat with sucrose supplemented with 1.6% Complex Tea Extract (HFHS + CTE), mice fed a high-fat/high-sucrose (HFHS) diet containing 58% kcal from fat with sucrose supplemented with 10^10^ cells/animal/day of BPL1^®^ HT administered in the drinking water (HFHS + BPL1^®^ HT), and mice fed a high-fat/high-sucrose (HFHS) diet containing 58% kcal from fat with sucrose supplemented with 1.6% Complex Tea Extract and with 10^10^ cells/animal/day of BPL1^®^ HT in the drinking water (HFHS + CTE + BPL1^®^ HT).

The commercial Complex Tea Extract used in this study consists of a proprietary blend of green and black tea leaves standardized to 10% total flavan-3-ols (monomeric and theaflavins) and 5% methylxanthines (caffeine, theobromine, and theophylline). It was directly obtained by water extraction of a proprietary blend of green and black tea leaves, as previously described [31]. The customized rodent diet was elaborated by the company Research Diets Inc. (New Brunswick, NJ, USA). The 1.6% of tea extract was added to the commercial high-fat/high-sucrose diet with reference D1233. Both HFHS and HFHS + CTE were isocaloric. The diet with reference D11112201 was used as the standard diet (chow).

Body weight and solid intake were measured once a week over the treatment period. The amount of BPL1^®^ HT in the drinking water was calculated every day depending on liquid intake to adjust the daily dose to 1010 cells/animal/day.

After 20 weeks of treatment, all animals were sacrificed by an overdose of sodium pentobarbital (100 mg/kg) and killed by decapitation after overnight fasting. After sacrifice, the blood was collected in tubes containing EDTA (1.5 mg/mL) and centrifuged at 3000 rpm for 20 min to obtain plasma. Liver, spleen, kidneys, adrenal glands, and the epididymal, retroperitoneal, subcutaneous, brown, and periaortic (PVAT) adipose tissue depots were weighed and stored at −80 °C for later analysis.

### 4.6. Plasma Measurements

Lipid profile (triglycerides, total cholesterol, low-density lipoprotein cholesterol (LDL-c) and high-density lipoprotein cholesterol (HDL-c)) were measured in plasma using commercial kits from Spin React S.A.U (Sant Esteve de Bas, Gerona, Spain). Glycerol plasma levels were measured using a commercial kit from Merck (Darmstadt, Germany), following the manufacturer’s instructions. Likewise, plasma concentrations of leptin, insulin and adiponectin were measured using ELISA kits (Merck Millipore, Dramstadt, Germany) following the manufacturer’s instructions. Sensitivity and intra-assay variations were 0.05 ng/mL and 1.76–3.01% for the leptin assay, 0.1 ng/mL and 1.92–7.64% for the insulin assay, and 0.2 ng/mL and 1.4–5.4% for the adiponectin assay. Blood glucose levels were measured after overnight fasting by tail vein puncture using the glucometer Glucocard™ G (Arkray Factory, Inc., Koji Konan-cho, Koka, Japan). The HOMA-IR index was calculated by the following formula: fasting glucose (mg/dL) × (fasting insulin (ng/mL)/405).

### 4.7. Analysis of Insulin Sensitivity in Metabolic Tissues

A total of 100 mg of liver and gastrocnemius explants were incubated in Dulbecco’s modified Eagle’s medium Gibco (DMEM/F-12; 1:1 mix; Invitrogen, Carlsbad, CA, USA) with 100 U/mL penicillin and 100 μg/mL streptomycin (Invitrogen, Carlsbad, CA, USA) in the presence/absence of insulin (10–6 M) (Sigma-Aldrich, St. Louis, MO, USA) at 37 °C in a 95% O2 and 5% CO_2_ incubator. After 15 min of incubation, all tissues were collected and stored at -80 °C for further Western blot (WB) analysis. For the WB, tissues were homogenized in 500 µL of lysis (RIPA) buffer and centrifugated at 14,000 rpm for 20 min at 4 °C. Total protein content was measured in the supernatant by Bradford method (Sigma-Aldrich, St. Louis, MO, USA). For each assay, 10 µL of total proteins were loaded in each well of 10% acrylamide SDS gels (Bio-Rad, Hercules, CA, USA) and separated by electrophoresis. The proteins were then transferred to polyvinylidene difluoride (PVDF) membranes (Bio-Rad, Hercules, CA, USA). Transfer efficiency was determined by Ponceau red dyeing (Sigma-Aldrich, St. Louis, MO, USA). Membranes were then blocked either with tris-buffered saline (TBS) containing 5% (*w*/*v*) non-fat dried milk (for non-phosphorylated proteins) or with 5% BSA (for phosphorylated proteins) and incubated with the appropriate primary antibody for Akt (1:1000; # 04-796, Merk Millipore, Dramstad, Germany) or p-Akt (Ser 473) (1:500; #9271, Cell Signaling Technology, Danvers, MA, USA) at 4 °C overnight. Membranes were subsequently washed and incubated with the secondary antibody conjugated with peroxidase (1:2000; Pierce, Rockford, IL, USA). Peroxidase activity was visualized by chemiluminescence and quantified by densitometry using BioRad Molecular Imager ChemiDoc XRS System (Hercules, CA, USA). For each sample, relative protein expression levels were calculated in relation to protein expression levels in mice fed with Chow.

### 4.8. Analysis of the p-eNOS/eNOS Ratio in Aortic Tissue

The p-eNOS/eNOS ratio was measured by Western blot as mentioned above using 1:1000 of the primary antibody for endothelial oxide nitric synthase (eNOS) (BD Bioscience, San José, CA, USA) and 1:500 of the primary antibody for phospho-endothelial oxide nitric synthase (p-eNOS) (1:500; Merck Millipore, Darmstadt, Germany).

### 4.9. Quantitative qRT-PCR

Total RNA was extracted from liver, gastrocnemius muscle, retroperitoneal adipose tissue, and aortic tissue, using the Tri-Reagent protocol [61]. cDNA was then synthesized from 1 µg of total RNA using a high-capacity cDNA reverse transcription kit (Applied Biosystems, Foster City, CA, USA). Assay-on-demand kits (Applied Biosystems, Foster City, CA, USA) were used for quantitative real-time polymerase chain reaction (qPCR). TaqMan Universal PCR Master Mix (Applied Biosystems, Foster City, CA, USA) was used for amplification according to the manufacturer’s instructions in a Step One System (Applied Biosystems, Foster City, CA, USA). The gene expression of interleukin-6 (Il-6) (Mm00446190_m1), interleukin-1 beta (Il-1β) (Mm00434228_m1), interleukin-10 (Il-10) (Mm01288386_m1), tumor necrosis factor-alpha (Tnf-α) (Mm00443258_m1), monocyte chemoattractant protein (Mcp-1) (Mm00441242_m1), glutathione reductase (Gsr) (Mm00439154_m1), NADPH oxidase-4 (Nox-4) (Mm00479246_m1), NADPH oxidase-1 (Nox-1) (Mm00549170_m1), superoxide dismutase 1 (Sod-1) (Mm01344233_g1), and Glutathione Peroxidase 3 (Gpx3) (Mm00492427_m1) were measured in liver, gastrocnemius muscle, retroperitoneal adipose tissue, and aortic tissue.

In addition, the gene expression of fatty acid synthetase (Fasn) (Mm00662319_m1), lipoprotein lipase (Lpl) (Mm00434764_m1), hormone-sensitive lipase (Hsl) (Mm00495359_m1), peroxisome proliferator-activator receptor-γ (Ppar-γ) (Mm00440940_m1), beta-3 adrenergic receptor (β3-Adr) (Mm02601819_g1), leptin receptor (Ob-r) (Mm00440181_m1), peroxisome proliferator-activated receptor gamma coactivator 1-alpha (Pgc-1α) (Mm01208835_m1), and uncoupling protein 1 (Ucp-1) (Mm01244861_m1) were analyzed in retroperitoneal adipose, and the gene expression of angiotensin receptor type-1 (At1r) (Mm00616371_m1), and angiotensin receptor type-2 (At2r) (Mm01341373_m1) were analyzed in aortic tissue. The values were normalized to the housekeeping gene Hypoxanthine Phosphoribosyltransferase 1 (Hprt-1) (Mm03024075_m1). To determine the relative expression levels, the ΔΔCT method was used as previously described [62]. Data were calculated considering 100% the gene expression levels in mice fed with Chow.

### 4.10. Histological Analysis

Retroperitoneal adipose tissue samples were fixed in 4% paraformaldehyde and embedded in paraffin wax. Then, 5 µm thick sections were obtained with a microtome and stained with Harris Hematoxylin and Eosin. Images were obtained with a Leica optical microscope with a 10× objective (Wetzlar, Germany). To determine adipocyte size, the area of each adipocyte was measured using FIJI for Windows 36 bit (NIH, Bethesda, MA, USA). For each sample, at least 30 randomly selected cells were measured in multiple fields of view, and the results were expressed as mean area per adipocyte.

For the analysis of hepatic lipid content, liver samples were frozen and embedded in OCT. Then, 5 µm thick cryostat sections were obtained and stained with Oil Red O (Bio-Optica, Milan, Italy) following the manufacturer’s instructions. Subsequently, staining with Hematoxylin Eosin was performed to stain the cell nucleus. Images were captured with a Leica optical microscope with a 10× objective (Wetzlar, Germany).

### 4.11. Measurement of Systolic and Diastolic Blood Pressure in Conscious Mice by Plethysmography

Systolic and diastolic blood pressure was measured twice weekly by transmission photoplethysmography using a blood pressure analysis system (BP-2000; Visitech Systems, Apex, NC, USA). For this purpose, an occlusion cuff was placed at the base of the tail of the mice which were immobilized in dark cages on a plate preheated to 37 °C. To ameliorate the stress associated with this technique, all mice were acclimated to the device at least three days before the first measurement. At least 10 measurements were performed for each mouse.

### 4.12. Vascular Reactivity

After sacrifice, the aorta was dissected and cut into 2 mm segments. Aorta rings were mounted in a 4 mL organ bath for isometric tension recording using a PowerLab data acquisition system (ADInstruments, Colorado Springs, CO, USA). In the organ bath, an optimal passive tension of 1 g was applied to each segment. Afterwards, they were let to equilibrate for 60–90 min. After equilibration, potassium chloride (KCl 100 mM, Merck Millipore, Burlington, MA, USA) was added to the organ bath to determine segment viability. Segments contracting less than 30mg after stimulation with KCl were discarded.

Thoracic aortic segments were used for vasodilatation studies. For this purpose, the segments were precontracted with the thromboxane analog U46619 10^−7.5^ M (Sigma-Aldrich, St. Louis, MO, USA). Afterwards, dose–response curves were performed in response to sodium nitroprusside (SNP; 10^−9^–10^−5^ M) and acetylcholine (Atch; 10^−9^–10^−4^ M) (Sigma-Aldrich, St. Louis, MO, USA). The response to a single dose of insulin (10^−6^ M) was also assessed. For the dose–response curves in response to Ach, aortic segments were preincubated in the presence/absence of the antioxidant apocynin (10^−6^ M) (Sigma-Aldrich, St. Louis, MO, USA) for 30 min. The % relaxation in response to each concentration of Ach or insulin was calculated considering the maximal relaxation (Emax) in response to NTP 10^−5^ M. This response was considered as 100% of relaxation.

Abdominal aortic segments were used for vasoconstriction studies. Dose–response curves were performed in response to the vasoconstrictors endothelin-1 (ET-1; 10^−10^–10^−6.5^ M), angiotensin-II (AngII; 10^−11^–10^−6^ M), and norepinephrine (NA; 10^−9^–10^−4^ M) (Sigma-Aldrich, St. Louis, MO, USA). Some aortic segments were preincubated with the nitric oxide synthase inhibitor L-NAME (10^−4^ M) (Sigma-Aldrich, St. Louis, MO, USA) for 30 min before the dose–response curve to NA. The contraction in response to each dose of the vasoconstrictors was represented as the % contraction to KCl.

### 4.13. Nitrite and Nitrate Determination

Then, 3 mm aorta segments of mice from the different experimental groups were incubated in 24-well plates using Dulbecco’s Modified Eagle’s Medium and Ham’s F12 medium (DMEM/F-12) with glutamine from Gibco (1:1 mix; Invitrogen, Carlsbad, CA, USA), supplemented with 100 U/mL penicillin and 100 μg/mL streptomycin (Invitrogen, Carlsbad, CA, USA). After 24h of incubation, the culture media was collected and the concentration of nitrites and nitrates (NO_2_^−^ and NO_3_^−^) as an index of NO release by the vascular endothelium (NO is a volatile gas that rapidly degrades into NO_2_^−^ and NO_3_^−^). The concentration of NO_2_^−^ and NO_3_^−^ was measured by the Gries method as previously described [63]. Briefly, 100 μL of vanadium chloride (Sigma-Aldrich, St. Louis, MO, USA) was added to 100 μL of culture medium in a 96-well plate. Immediately afterward, the Griess reagent (a 1:1 mixture of 1% sulfanilamide and 0.1% naphthylethylenediamine dihydrochloride, both from Merck Millipore, Darmstadt, Germany) was added to each well and incubated at 37 °C for 30 min. The absorbance was then measured at 540 nm. NO_2_^−^ and NO_3_^−^ concentrations were determined using a NaNO_2_ standard curve and expressed in μM.

### 4.14. Analysis of Gut Microbiome Composition

Fecal samples were collected at the end of the study. Microbial DNA was isolated from stool samples using the QIAsymphony PowerFecal Pro DNA Kit (Qiagen, Hilden, Germany). The V3–V4 hypervariable region of the 16S rRNA gene was amplified from genomic DNA using primers 341F (CCTACGGGNGGCWGCAG) and 805R (GACTACHVGGG TATCTAATCC). The 16S rRNA libraries were quantified via fluorometry using the Quant-iT™ Picofreen™ dsDNA Assay Kit (Thermofisher, Waltham, MA, USA). Prior to sequencing on the MiSeq platform (Illumina, San Diego, CA, USA) with a paired-end read configuration of 300 cycles, the libraries were pooled. The size and quantity of the pooled libraries were evaluated using the Bioanalyzer 2100 (Agilent, Santa Clara, CA, USA) and the Library Quantification Kit for Illumina (Kapa Biosciences, Oslo, Norway), respectively. The PhiX Control library (v3) (Illumina) was added to the amplicon library. Image analysis, base calling, and quality assessment of the data were conducted using the MiSeq instrument and its MiSeq Control Software (MCS v2.6.2.1). Forward and reverse reads were merged using the BBMerge tool from the BBMap V.39 software (https://sourceforge.net/projects/bbmap/; accessed on 13 January 2025 accessed on 13 January 2025). To minimize bias during annotation, amplification primers were trimmed with Cutadapt v4.9 (https://journal.embnet.org/index.php/embnetjournal/article/view/200; accessed on 13 January 2025).

A quality filtering step was applied to exclude low-quality sequences using the Reformat tool from BBMap V.39 software. Sequences shorter than 200 nucleotides were excluded from the analysis, and bases at the ends of sequences with a Phred score below Q20 were removed. Sequences with an average quality score below Q20 across their entire length were also discarded. The reads were processed using the DADA2 v1.26 software (https://github.com/benjjneb/dada2; accessed on 13 January 2025)with the ‘denoise-single’ command [64]. Error rates were calculated from a subset of reads using the ‘learnErrors’ function, and sample inference was performed with the ‘dada’ function to generate Amplicon Sequence Variants (ASVs). Chimeric ASVs were removed using the ‘removeChimeraDenovo’ function. Taxonomic annotation of ASVs was carried out using the NCBI 16S rRNA database version 2024 and the blastn tool (version 2.2.29+) [65]. ASVs with less than 97% identity were reclassified using the NBAYES algorithm [66] within the QIIME2 platform v2024.5 [67]. The NBAYES classifier was trained on the V3–V4 regions of the 16S rRNA gene using the SILVA v.138 database [68]. ASVs classified within the bacterial kingdom and present at a relative frequency of at least 0.001% in a minimum of three samples were retained for subsequent bioinformatics analysis.

For alpha diversity analysis, data were normalized using a rarefaction technique provided by the Phyloseq R package v1.46 [69]. Metrics such as Shannon, Simpson, and richness indexes were calculated using the vegan R package v2.6-6.1 (https://github.com/vegandevs/vegan; accessed on 13 January 2025). An ANOVA followed by a *t*-test or Kruskal–Wallis test followed by the Wilcoxon test was performed to assess differences between groups for parametric or non-parametric data, respectively.

Differential abundance of taxa was analyzed using the DESeq2 R package v1.42.0 [70], with normalization based on the ‘Relative Log Expression’ method. Scaling factors were calculated using the ‘EstimateSizeFactors’ function, which applies the median ratio between taxa abundances and the geometric mean. The ‘PosCounts’ method was used to account for taxa with multiple zeros across most samples, a common occurrence in metagenomic data. Taxa were considered differentially abundant if they had a Benjamini–Hochberg (BH) adjusted *p*-value < 0.05 and were present in at least 50% of samples within one of the compared groups. The MaAslin2 R package v1.4 [71] was utilized to explore the association between microbial abundances and clinical and experimental variables. A linear model test was applied to each variable, treating the variable as a fixed effect. Microbial taxa counts were normalized using the DESeq2 normalization method, followed by log transformation of the normalized counts. Only taxa present in more than 20% of the samples were included in the analysis.

Heatmaps were created to summarize bacteria taxa differential abundance and the association between taxa abundance and clinical and experimental variables using the ComplexHeatmap R package v2.11.1 [72].

### 4.15. Statistical Analysis

Statistical analysis was performed by one-way ANOVA followed by Bonferroni’s post hoc test or t-student for the experiments of explant incubation in the presence/absence of insulin using the GraphPad Prism V.7.04 statistical software (GraphPad Software, Inc., San Diego, CA, USA). Data are expressed as mean values ± standard error of the mean (SEM). Comparisons with *p* < 0.05 were considered statistically significant.

## Figures and Tables

**Table 1:** Bioactive composition (%, dry basis) of Tea Complex (CTE) throughout different in vitro digestion steps analyzed by HPLC.

Bioactive Components (%, Dry Basis)	CTE Control	CTE Digested	Decrease (Folds)
Theobromine	0.44 ± 0.15	0.12 ± 0.07	3.67
Gallocatechin	0.71 ± 0.54	0.04± 0.03	17.75
Theophyllyne	0.05 ± 0.01	0.02 ± 0.00	2.50
Epigallocatechin	2.05 ± 1.93	0.04 ± 0.04	51.25
Catechin	0.41 ± 0.13	0.14 ± 0.11	2.93
Caffeine	6.55 ± 0.51	1.81 ± 0.66	3.62
Epicatechin	1.30 ± 0.54	0.31 ± 0.20	4.19
EGCg	9.29 ± 1.14	0.11 ± 0.17	84.45
Gallocatechin-3-gallate	1.14 ± 0.40	0.06 ± 0.07	19.00
Epicatechin-3-gallate	2.96 ± 1.41	0.50 ± 0.46	5.92
Catechin-3-gallate	1.60 ± 2.00	0.09 ± 0.06	17.78
Theaflavins	0.43 ± 0.12	0.12 ± 0.01	3.58
Total flavan-3-ols	19.46 ± 3.92	1.23 ± 0.98	15.82
Total xanthines	7.02 ± 0.60	1.93 ± 0.73	3.64

**Table 2:** Body weight at sacrifice. Body weight gain during treatment. Total caloric intake and weights of organs and tissues of mice fed a standard chow (Chow), a high-fat diet/sucrose diet (HFHS), a high-fat diet/sucrose diet supplemented with Complex Tea Extract (HFHS + CTE), a high-fat diet/sucrose diet supplemented with BPL1^®^ HT (HFHS + BPL1^®^ HT), and mice fed a high-fat diet/sucrose diet supplemented with Complex Tea Extract and BPL1^®^ HT (HFHS + CTE + BPL1^®^ HT).

Weight	Chow	HFHS	HFHS + CTE	HFHS + BPL1^®^ HT	HFHS + CTE + BPL1^®^ HT
Body weight (g)	31.0 ± 0.9	48.4 ± 22 ***	39.3 ± 2 ***^##^	39.2 ± 1.3 ***^##^	34 ± 2.7 ^###^
Body weight gain (g)	3.4 ± 0.5	19.5 ± 2 ***	12.7 ± 1.7 ***^#^	11.9 ± 1.5 ***^##^	5.6 ± 1.9 ^###$$&^
Total caloric intake (kcal)	1670 ± 75	2012 ± 90 **	1757 ± 78.6 *^#^	1661 ± 49.2 ^#^	1678 ± 107 ^#^
Heart (mg/cm)	85.5 ± 3.1	113 ± 9.7 *	89.2 ± 6.7 ^#^	82.9 ± 13.6 ^#^	63.7 ± 12.3 ^##$^
Spleen (mg/cm)	33.8 ± 1.8	65.6 ± 5.7 ***	42.8 ± 2.1 **^###^	42.5 ± 2.2 *^###^	36.4 ± 2.4 ^###$&^
Liver (mg/cm)	583.4 ± 20	920.5 ± 175 *	583.3 ± 49.3 ^#^	575.6 ± 24.1 ^#^	527 ± 50.9 ^#^
Epididymal visceral adipose tissue (mg/cm)	364 ± 52	1460 ± 61.5 ***	1138 ± 139.5 ***^#^	1226 ± 126 ***^#^	654 ± 168 ^###$&^
Retroperitoneal visceral adipose tissue (mg/cm)	168.8 ± 33	674.8 ± 72 ***	481 ± 64.8 ***^#^	513 ± 54.1 ***^#^	247 ± 92 ^##$&^
Lumbar subcutaneous adipose tissue (mg/cm)	122.8 ± 17	8771.4 ± 106 ***	488 ± 74.1 ***^##^	480 ± 57.3 ^##^	237 ± 73.5 ^###$&^
Interscapular brown adipose tissue (mg/cm)	58 ± 7.1	137.7 ± 16.6 ***	85.5 ± 10.1 *^#^	85.3 ± 7.2 ^##^	58.9 ± 8.1 ^###$&^
Periaortic adipose tissue (mg/cm)	5.9 ± 0.7	12.2 ± 1.2 ***	8 ± 0.7 *^##^	8.7 ± 0.7 **^#^	5.9 ± 0.5 ^###$&&^
Kidneys (mg/cm)	171 ± 6.8	221.4 ± 10.8 ***	183 ± 8.4 ^#^	188 ± 8.9 ^#^	180 ± 6.9 ^##^
Adrenal glands (mg/cm)	1.5 ± 0.1	2.4 ± 0.3 **	1.7 ± 0.2 ^#^	1.6 ± 0.1 ^#^	1.7 ± 0.07 ^#^
Brain (mg/cm)	251 ± 1.3	257 ± 2 *	245 ± 2.8 *^##^	250 ± 2.83 ^#^	250 ± 1.6 ^##^
Gastrocnemius (mg/cm)	79.2± 1.2	86.6 ± 1.44 ***	80.8 ± 2.1 ^#^	79.6 ± 1.3 ^##^	77.8 ± 1.6 ^###^

Data are represented as mean value ± SEM; *n* = 8–10 mice/group. * *p* < 0.05 vs. Chow; ** *p* < 0.01 vs. Chow; *** *p* < 0.001 vs. Chow; ^#^ *p* < 0.05 vs. HFHS; ^##^ *p* < 0.01 vs. HFHS; ^###^ *p* < 0.001 vs. HFHS; ^$^ *p* < 0.05 vs. HFHS + CTE; ^$$^ *p* < 0.01 vs. HFHS + CTE. ^&^ *p* < 0.05 vs. HFHS + BPL1^®^ HT; ^&&^ *p* < 0.01 vs. HFHS + BPL1^®^ HT. Red color represents differences between mice fed with the HFHS diet in comparison to mice fed with chow; green color represents a significant improvement of supplemented mice fed with the HFHS diet vs. non-supplemented mice fed with HFHS; purple color represents a synergistic effect between CTE and BPL1^®^ HT.

**Table 3:** Plasma parameters: total cholesterol. LDL-cholesterol. HDL-cholesterol. Triglycerides. Glycaemia. Insulin. Adiponectin. Leptin. Homeostatic Model Assessment of Insulin Resistance (HOMA-IR) and glycerol of mice fed a standard chow (Chow), mice fed a high-fat diet/sucrose diet (HFHS), mice fed a high-fat diet/sucrose diet supplemented with Complex Tea Extract (HFHS + CTE), mice fed a high-fat diet/sucrose diet supplemented with BPL1^®^ HT (HFHS + BPL1^®^ HT), and mice fed a high-fat diet/sucrose diet supplemented with Complex Tea Extract and BPL1^®^ HT (HFHS + CTE + BPL1^®^ HT).

	Chow	HFHS	HFHS + CTE	HFHS + BPL1^®^ HT	HFHS + CTE + BPL1^®^ HT
Total Cholesterol (mg/dL)	106 ± 6.5	211 ± 17.7 ***	209 ± 9.3 ***	177.48 ± 8.32 ***^#$^	146 ± 23.8 ^#$^
LDL-cholesterol (mg/dL)	11.2 ± 0.7	18.6 ± 3.1 *	14.6 ± 1.4 *	15.5 ± 2.2 *	10.7 ± 1.3 ^#$&^
HDL-cholesterol (mg/dL)	17.3 ± 1.1	31.9 ± 4 **	30.7 ± 0.8 ***	26.8 ± 1.7 ***	25.8 ± 3.2 *
Triglycerides (mg/dL)	42.9 ± 4.5	75.8 ± 8.5 **	72.2 ± 7.7 **	93.9 ± 6.9 ***^$^	62.8 ± 10.6 ^#&^
Glycaemia (mg/dL)	118 ± 9.4	147 ± 9.45 *	148 ± 9.4 *	134 ± 10.6	120 ± 4.7 ^##$$^
Insulin (ng/mL)	0.86 ± 0.1	5.1 ± 1.4 **	2 ± 0.3 **^#^	2.1 ± 0.3 **^#^	2 ± 0.5 *^#^
Adiponectin (ng/mL)	10,916 ± 502	9246 ± 642 *	10,633 ± 542	10,296 ± 549	11,506 ± 1031 ^#^
Leptin (ng/mL)	2.6 ± 0.9	33.9 ± 3.1 ***	19.6 ± 3.5 ***^#^	17.9 ± 2.8 ***^##^	7.9 ± 4.1 ^###$&^
HOMA-IR	0.3 ± 0.1	1.9 ± 0.5 **	0.87 ± 0.2 **^#^	0.77 ± 0.1 **^#^	0.47 ± 0.1 ^##$&^
Glycerol (mg/dL)	1.2 ± 0.1	1.7 ± 0.0 ^1^**	1.4 ± 0.04 *^##^	1.9 ± 0.07 ***^$$$^	1.06 ± 0.2 ^##$&&^

Data are represented as mean value ± SEM; *n* = 8–10 mice/group. * *p* < 0.05 vs. Chow; ** *p* < 0.01 vs. Chow; *** *p* < 0.001 vs. Chow; ^#^ *p* < 0.05 vs. HFHS; ^##^ *p* < 0.01 vs. HFHS; ^###^ *p* < 0.001 vs. HFHS; ^$^ *p* < 0.05 vs. HFHS + CTE; ^$$^ *p* < 0.01 vs. HFHS + CTE; ^$$$^ *p* < 0.001 vs. HFHS + CTE; ^&^ *p* < 0.05 vs. HFHS + BPL1^®^ HT; ^&&^ *p* < 0.01 vs. HFHS + BPL1^®^ HT. Red color represents differences between mice fed with the HFHS diet in comparison to mice fed with chow; green color represents a significant improvement of supplemented mice fed with the HFHS diet vs. non-supplemented mice fed with HFHS; purple color represents a synergistic effect between CTE and BPL1^®^ HT.

**Table 4:** Heart rate, diastolic arterial pressure, and systolic arterial pressure of mice fed a standard chow (Chow), mice fed a high-fat diet/sucrose diet (HFHS), mice fed a high-fat diet/sucrose diet supplemented with Complex Tea Extract (HFHS + CTE), mice fed a high-fat diet/sucrose diet supplemented with BPL1^®^HT (HFHS + BPL1^®^ HT), and mice fed a high-fat diet/sucrose diet supplemented with Complex Tea Extract and BPL1^®^ HT (HFHS + CTE + BPL1^®^ HT).

	Chow	HFHS	HFHS + CTE	HFHS + BPL1^®^ HT	HFHS + CTE + BPL1^®^ HT
Heart rate (bpm)	654.7 ± 16.0	709.3 ± 13.3 ***	712.4 ± 10.6 ***	702.8 ± 12.3 ***	705.8 ± 13.0 ***
Diastolic blood pressure (mmHg)	55.3 ± 3.8	53.5 ± 4.0	53.4 ± 4.2	53.0 ± 4.0	54.2 ± 3.9
Systolic blood pressure (mmHg)	119.1 ± 3.0	125.5 ± 3.8 ***	117.8 ± 3.2 ^###^	121.3 ± 3.2 ^##^	118.3 ± 3.1 ^###&^

Data are represented as mean value ± SEM; *n* = 8–10 mice/group. *** *p* < 0.001 vs. Chow; ^##^ *p* < 0.01 vs. HFHS; ^###^ *p* < 0.001 vs. HFHS; ^&^ *p* < 0.05 vs. HFHS + BPL1^®^ HT. Red color represents differences between mice fed with the HFHS diet in comparison to mice fed with chow; green color represents a significant improvement of supplemented mice fed with the HFHS diet vs. non-supplemented mice fed with HFHS.

**Table 5:** Concentration of nitrites and nitrates released from aorta segments of mice fed a standard chow (Chow), mice fed a high-fat diet/sucrose diet (HFHS), mice fed a high-fat diet/sucrose diet supplemented with Complex Tea Extract (HFHS + CTE), mice fed a high-fat diet/sucrose diet supplemented with BPL1^®^ HT (HFHS + BPL1^®^ HT), and mice fed a high-fat diet/sucrose diet supplemented with Complex Tea Extract and BPL1^®^ HT (HFHS + CTE + BPL1^®^ HT).

	Chow	HFHS	HFHS + CTE	HFHS + BPL1^®^ HT	HFHS + CTE + BPL1^®^ HT
NO_2_^−^ + NO_3_^−^ (μM)	28.5 ± 1.4	25.6 ± 0.3 *	28.3 ± 2.9	27.5 ± 0.6	26.7 ± 0.6

Data are represented as mean value ± SEM; *n* = 8–10 mice/group. * *p* < 0.05 vs. Chow.

## Data Availability

The original data are not publicly available due to confidentiality restrictions, as the results are the property of ADM and are currently being reviewed for potential patent protection.

## Supplementary Materials

The following supporting information can be downloaded at: https://www.mdpi.com/article/10.3390/ijms27020680/s1.

## Abbreviations

The following abbreviations are used in this manuscript:

ACE2	Angiotensin converting enzyme 2
Ach	Acetylcholine
AngII	Angiotensin II
At1r	Angiotensin receptor type-1
At2r	Angiotensin receptor type-2
BPL1^®^ HT	Heat-treated postbiotic
CTE	ComplexTea Extract
EGCG	(-)-epigallocatechin-3-gallate
eNOS	Endothelial nitric oxide synthase
ET-1	Endothelin-1
Fasn	Fatty acid synthetase
Gpx3	Glutathione Peroxidase 3
Gsr	Glutathione reductase
HDL-c	High-density lipoprotein cholesterol
HFHS	High-fat/high-sucrose
Hprt-1	Hypoxanthine Phosphoribosyltransferase 1
Hsl	Hormone-sensitive lipase
Il-10	Interleukin-10
Il-1β	Interleukin-1 beta
Il-6	Interleukin-6
ISAPP	International Scientific Association of Probiotics and Prebiotics
LDL-c	Low-density lipoprotein cholesterol
Lpl	Lipoprotein lipase
Mcp-1	Monocyte chemoattractant protein
MetS	Metabolic syndrome
NA	Noreppinephrine
NO	Nitric oxide
Nox-1	NADPH oxidase-1
Nox-4	NADPH oxidase-4
NTP	Sodium nitroprusside
Ob-r	Leptin receptor
Pgc-1α	Peroxisome proliferator-activated receptor gamma coactivator 1-alpha
PI3K/Akt	Phosphatidylinositol 3-kinase/protein kinase B
Ppar-γ	Peroxisome proliferator-activator receptor-γ
RAAS	Renin–angiotensin–aldosterone system
ROS	Reactive oxygen species
Sod-1	Superoxide dismutase 1
Tnf-α	Tumor necrosis factor-alpha
Ucp-1	Uncoupling protein 1
β3-Adr	Beta-3 adrenergic receptor
