# Supplementary material for: Enhanced Effects of Complex Tea Extract and the Postbiotic BPL1® HT on Ameliorating the Cardiometabolic Alterations Associated with Metabolic Syndrome in Mice
Source: Int J Mol Sci. 2026 Jan 9;27(2):680. doi: 10.3390/ijms27020680 (PMC12840985; doi:10.3390/ijms27020680)
Supplement: Supplementary file 1 [file ijms-27-00680-s001.zip › ijms-3931029-supplementary.pdf]

# Supplementary Materials

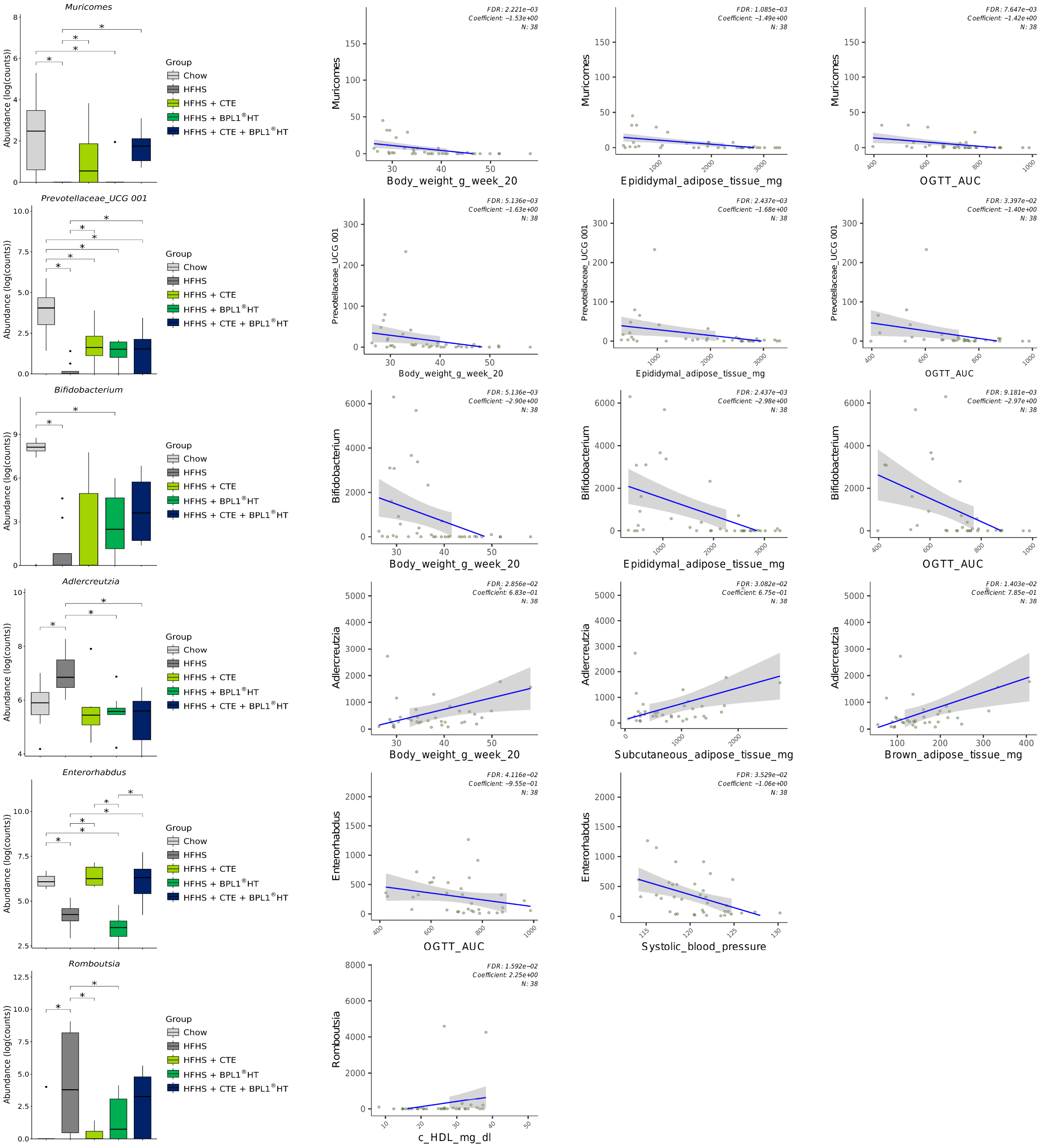


Figure S1. Abundance of bacterial genera altered by a high-fat diet/sucrose diet and modulated by the supplementation of Complex Tea Extract, BPL1® HT or blend. The first column shows the boxplots of abundance (logarithm) of bacteria genera in each group. The second, third, and fourth column shows the linear relationship between genera abundance and clinical features. The coefficients and statistical significance corrected by false discovery rate (FDR) were calculated by Maaslin2. * in boxplots mean

differential abundance in taxa between groups calculated by DESeq2, corrected by FDR and only if taxa are present in at least 50% of the samples of one of the compared groups.

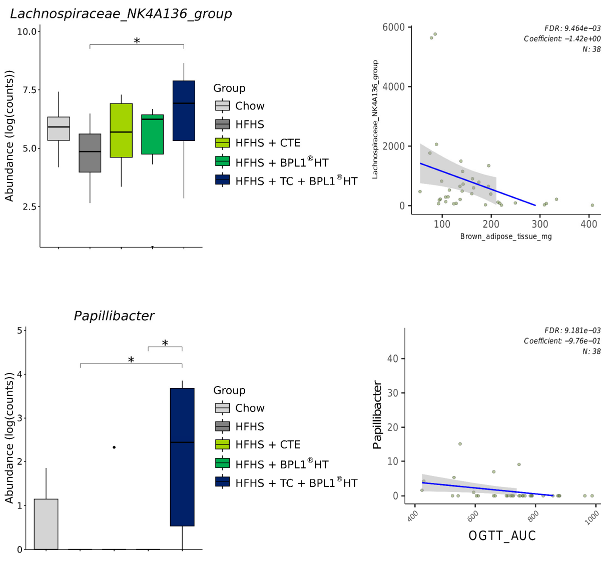


Figure S2. Abundance of bacterial genera modulated only by the supplementation of a blend of Complex Tea Extract and BPL1® HT during high-fat diet/sucrose diet (HFHS + CTE + BPL1® HT) associated with clinical parameters of metabolic syndrome. The first column shows the boxplots of abundance (logarithm) of bacteria genera in each group. The second shows the linear relationship between genera abundance and clinical features. The coefficients and statistical significance corrected by false discovery rate (FDR) were calculated by Maaslin2. * in boxplots mean differential abundance in taxa between groups calculated by DESeq2, corrected by FDR and only if taxa are present in at least 50% of the samples of one of the compared groups.

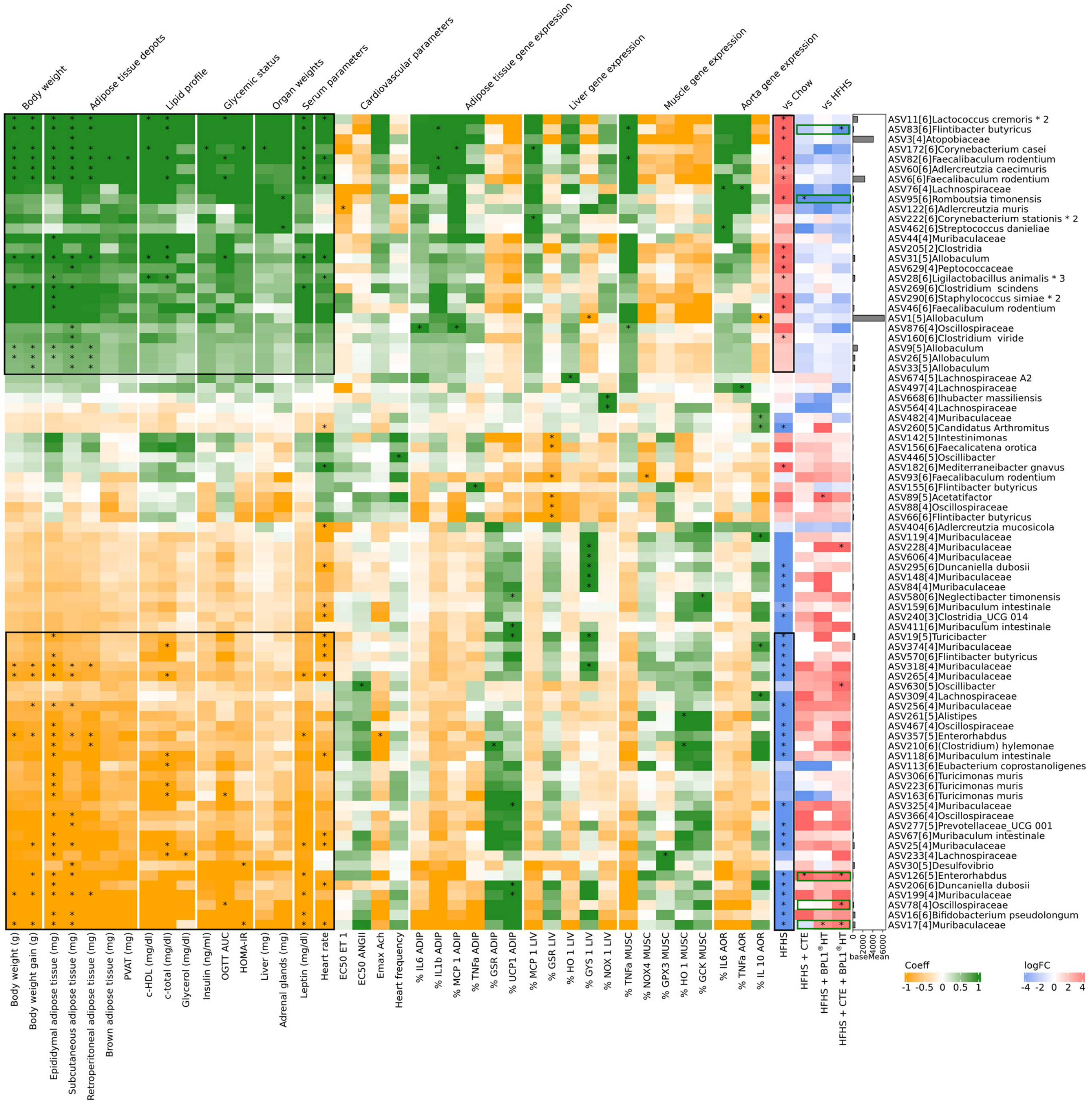


Figure S3. Association heatmaps showing the Maaslin2 Coefficient (Coeff) between clinical variables, gene expression and protein expression and gut ASV abundances. Left heatmap in green and orange: green color means the feature is directly associated with taxa abundance, while orange color is inversely associated. Right heatmap in red and blue: red means that the taxon is over-represented in the first group of the comparison, while blue means that the taxon is under-represented in the first group. Black boxes highlight taxa associated with several clinical parameters of metabolic syndrome and altered by high-fat diet/sucrose diet (HFHS) compared to standard chow (Chow); green boxes highlight the taxa that were altered by HFHS compared to Chow and modulated by the supplementation with Complex Tea Extract (HFHS+CTE), BPL1® HT (HFHS+ BPL1® HT) or the blend Complex Tea Extract and BPL1® HT (HFHS+CTE+ BPL1® HT). * adj. $p < 0.05$ in left heatmap means the feature is significantly associated with

taxa abundance. * adj. $p < 0.05$ in right heatmap means the differential abundance between groups and the taxon is present in at least 50% of samples of one of the compared groups. BaseMean bar plots show the mean abundances of each ASV.

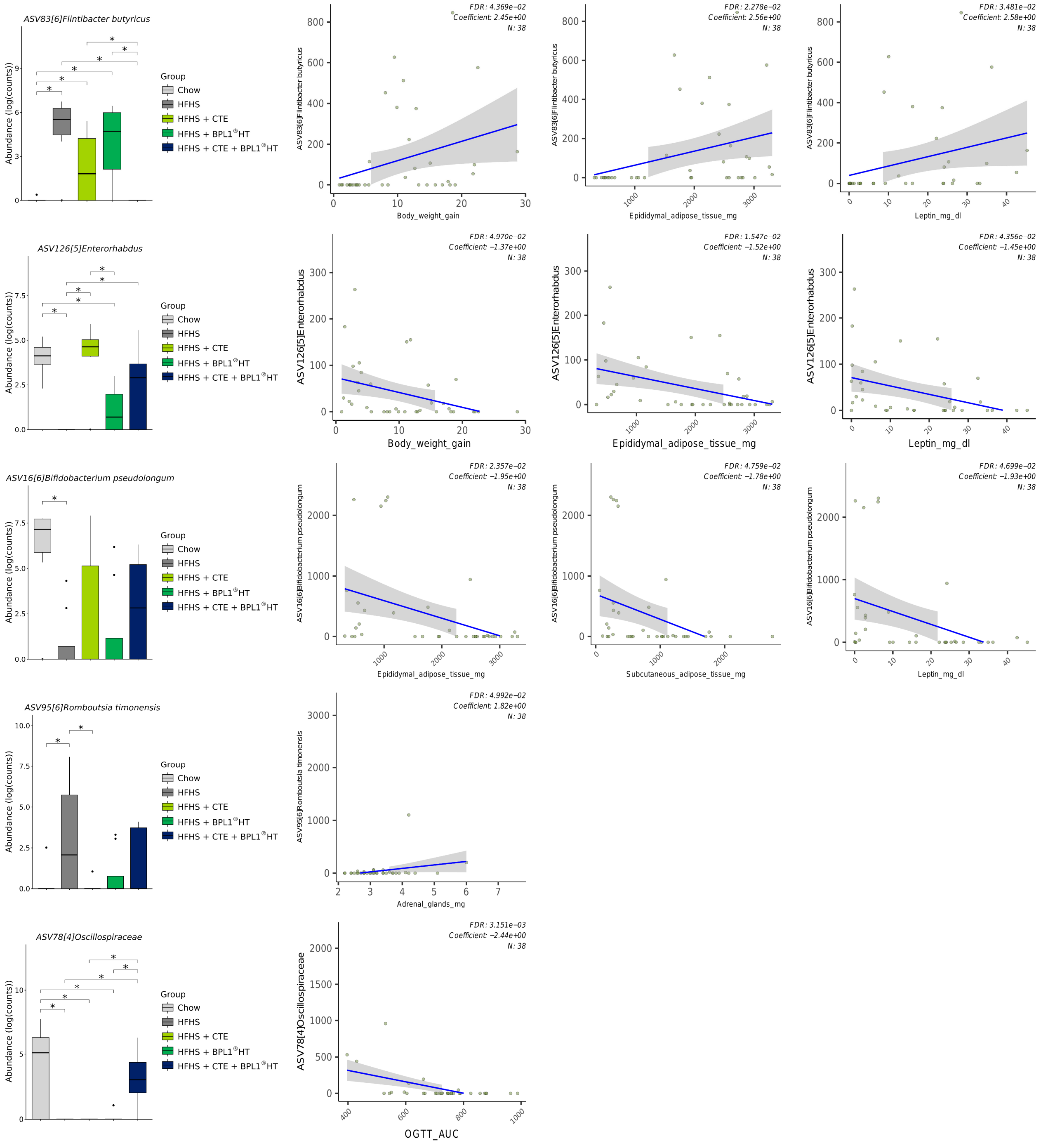


Figure S4. Abundance of bacterial ASV altered by a high-fat diet/sucrose diet and modulated by the supplementation of Complex Tea Extract, BPL1® HT or blend. The first column shows the boxplots of abundance (logarithm) of ASV in each group. The second, third, and fourth column shows the linear relationship between ASV abundance and clinical features. The coefficients and statistical significance corrected by false discovery rate (FDR) were calculated by Maaslin2. * in boxplots mean differential abundance in taxa between groups calculated by DESeq2,

corrected by FDR and only if taxa are present in at least 50% of the samples of one of the compared groups.
